# Targeting the SIRT6–TDO2/KYNA–mTOR axis rescues synaptic and cognitive deficits in fetal growth restriction offspring

**DOI:** 10.1038/s12276-026-01739-7

**Published:** 2026-06-11

**Authors:** Shujuan Chang, Wen Chen, Wei Zhu, Nana Liu, Yuhang Wang, Jianguo Li, Jiuhong Kang

**Affiliations:** 1 Clinical and Translational Research Center of Shanghai First Maternity and Infant Hospital, Shanghai Key Laboratory of Maternal Fetal Medicine, Frontier Science Center for Stem Cell Research, National Stem Cell Translational Resource Center, School of Life Sciences and Technology, Tongji University, Shanghai, China; 2 Department of Medicine, Jinggangshan University, Ji’an, China

**Keywords:** Epigenetics in the nervous system, Neurological disorders

## Abstract

Fetal growth restriction (FGR), a major perinatal complication, is causally linked to lifelong cognitive deficits in offspring; however, its underlying mechanisms remain poorly defined. Here, the SIRT6–TDO2/KYNA–mTOR axis is identified as a critical mediator of synaptic dysfunction and cognitive deficits in FGR offspring. Hippocampal excitatory neurons in FGR mice exhibit markedly reduced SIRT6 expression, and SIRT6 conditional knockout in CaMKIIα⁺ neurons (*Sirt6* cKO) recapitulates FGR-induced synaptic and cognitive impairments. Mechanistically, SIRT6 governs synaptic plasticity and cognition via its histone deacetylase activity, independent of its ADP-ribosyltransferase function. SIRT6 deficiency increases histone H3K9 acetylation at the *Tdo2* promoter, enhancing kynurenine pathway flux and leading to pathological accumulation of hippocampal kynurenic acid (KYNA). Elevated KYNA suppresses AKT/mTOR/p70S6K1 signaling, disrupting synaptic protein synthesis. Strikingly, pharmacological TDO2 blockade, neuronal TDO2 knockdown or mTOR activation reverses synaptic and cognitive deficits in *Sirt6* cKO mice. Crucially, hippocampal SIRT6 overexpression in FGR mice normalizes KYNA levels, reactivates mTOR signaling, and restores synaptic plasticity and cognitive performance. These findings uncover a neurodevelopmental axis wherein neuronal SIRT6 deficiency dysregulates tryptophan metabolism to impair synaptic plasticity, identifying actionable targets for treating FGR-induced cognitive disorders.

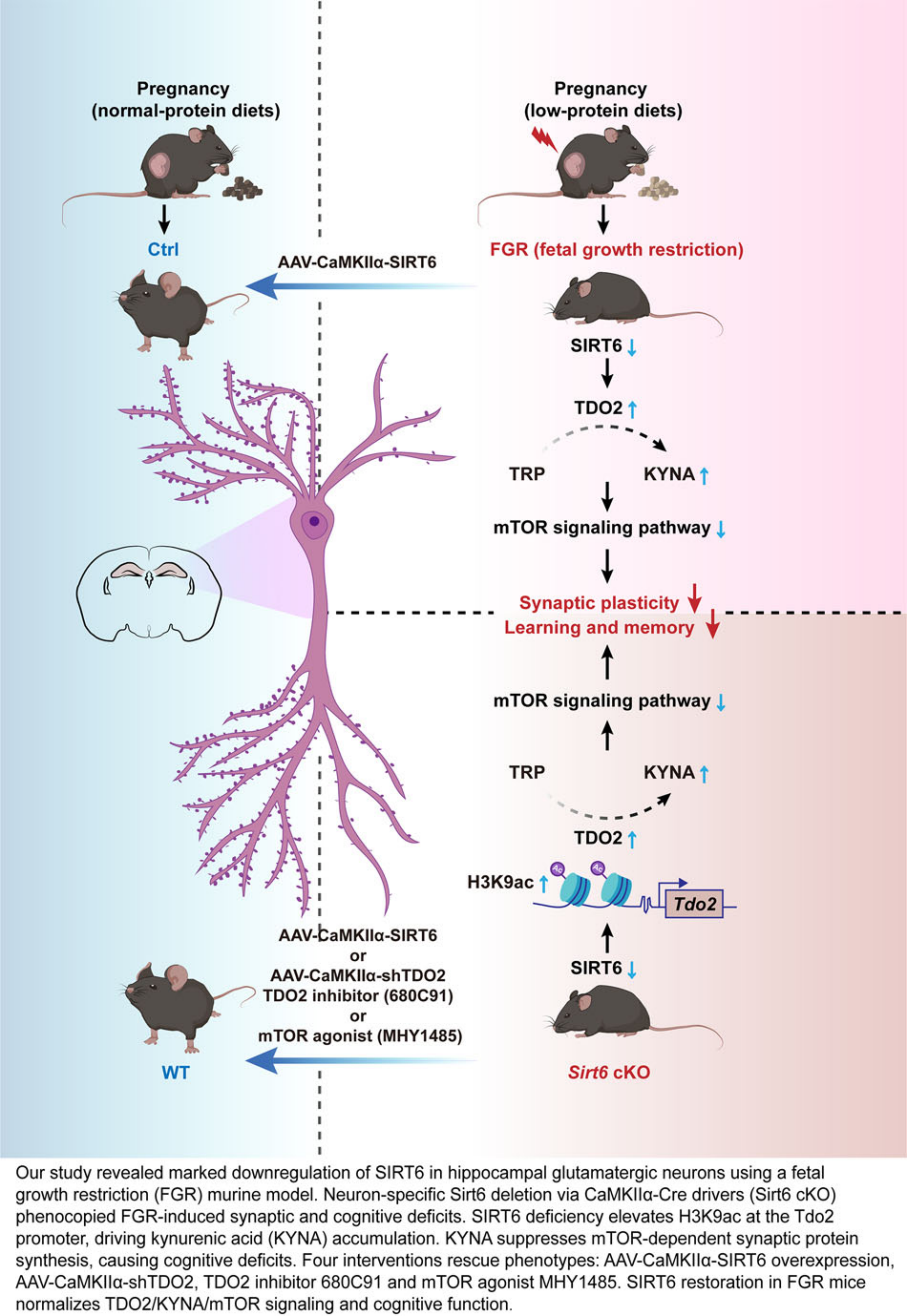

## SIRT6 deficiency disrupts synaptic function in FGR offspring

Fetal growth restriction (FGR), a condition affecting about 5% of pregnancies, is linked to significant neurodevelopmental issues and cognitive deficits in offspring. This study explores the role of the SIRT6 protein in hippocampal neurons, revealing its critical involvement in synaptic plasticity and cognitive function. Researchers used a mouse model to demonstrate that FGR leads to reduced SIRT6 expression, impairing synaptic plasticity and cognition by disrupting tryptophan metabolism, specifically through the TDO2/KYNA pathway. They used techniques such as CaMKIIα-Cre-mediated neuronal SIRT6 knockout and overexpression, behavioral tests, and biochemical analyses to elucidate these mechanisms. Findings show that SIRT6 deficiency upregulates TDO2, increasing neurotoxic KYNA levels, which suppress mTOR signaling, crucial for synaptic protein synthesis. Overexpressing SIRT6 or inhibiting TDO2 restored cognitive function, highlighting potential therapeutic targets for FGR-induced cognitive disorders. Future research could explore SIRT6 activators or KYNA-lowering compounds to mitigate these deficits.

This summary was initially drafted using artificial intelligence, then revised and fact-checked by the author.

## Introduction

Fetal growth restriction (FGR), clinically defined as estimated fetal weight below the 10th percentile for gestational age, represents a global pregnancy complication affecting ~5% of gestations^1^. Ranked as the second leading cause of perinatal mortality, this condition demonstrates strong associations with neurodevelopmental disorders in later life^2^. A robust body of epidemiological evidence has established persistent adverse cognitive outcomes in FGR offspring, manifesting as impaired learning and memory abilities, reduced intelligence quotient, and academic challenges that persist from early childhood through adolescence into adulthood^3–8^. Placental insufficiency is a major cause of FGR, and the resulting fetal nutrient and oxygen deficiency suggests that metabolic dysregulation has a central role in the chronic diseases induced by FGR^9^. However, the molecular mechanisms underlying metabolic dysregulation-induced cognitive impairment in offspring remain poorly understood. Given that FGR is generally diagnosed in mid-to-late gestation and effective prenatal interventions remain limited, elucidating neurodevelopmental abnormalities and establishing postnatal therapeutic strategies in FGR offspring hold critical scientific and clinical value for ameliorating long-term cognitive deficits.

Hippocampal synaptic loss and dysfunction emerge as neuropathological hallmarks of FGR-associated cognitive deficits^3,5,10–12^. Synaptic plasticity, the neurobiological substrate for learning and memory, is tightly regulated by epigenetic mechanisms that coordinate gene expression^13^. The NAD⁺-dependent sirtuin family (SIRT1–SIRT7) comprises histone deacetylases that govern both metabolic homeostasis and cognitive function by modulating chromatin structure and gene transcription^14–16^. Among them, nuclear-enriched SIRT6 is particularly notable: its deletion results in postnatal growth retardation and weight loss in mice^17,18^, and biallelic inactivating mutations in human SIRT6 recapitulate the FGR phenotype^19^. SIRT6 governs neuronal differentiation and neurodevelopment, with loss of function inducing neurodevelopmental deficits in primates^19,20^ and overexpression enhancing neurogenesis in murine models^21^. Neural stem-cell-specific SIRT6 deletion in mice impairs memory^22^, whereas astrocyte-specific deletion elicits antidepressant-like effects without affecting cognition^23^. These findings suggest that SIRT6 may have a previously unrecognized critical role in FGR-induced neurological abnormalities. Consequently, investigating the mechanisms by which SIRT6 regulates synaptic plasticity will help elucidate how metabolic dysregulation contributes to cognitive impairment in FGR offspring.

Clinical multisample analyses (placenta, umbilical cord, and maternal/neonatal blood) consistently demonstrate dysregulated tryptophan (TRP) metabolism in FGR^24–26^. As TRP derivatives critically modulate neurodevelopmental programming and cognitive circuitry^27^, their altered homeostasis raises mechanistic concerns. In the central nervous system, >95% of L-TRP undergoes kynurenine pathway (KP) conversion, generating neuroactive metabolites such as kynurenic acid (KYNA). TRP undergoes rate-limiting conversion by indoleamine 2,3-dioxygenase (IDO) or tryptophan 2,3-dioxygenase (TDO) to generate kynurenine (KYN), which is subsequently converted by kynurenine aminotransferases (KATs) to form KYNA^28,29^. Elevated brain KYNA levels have been linked to cognitive dysfunction, including impairments in working memory, spatial memory, and attentional processing^30–32^. Conversely, targeted suppression of KYNA biosynthesis (genetic/pharmacological) rescues cognitive phenotypes in rodent and primate models^33–36^. The mechanisms by which FGR epigenetically reprograms key nodes of TRP-KP metabolism, and whether this pathway mediates neurodevelopmental abnormalities in FGR offspring, remain unclear.

Metabolic disturbances frequently disrupt cerebral homeostasis by modulating nutrient-sensing signaling pathways, with the mTOR pathway operating as a master regulatory nexus that synchronizes metabolic signals with protein synthesis^37,38^. This kinase network responds to a broad range of inputs, including amino acids and their metabolites, energy status, and growth factors^39^. Through its downstream effectors in the AKT–mTOR–p70S6K1 axis, mTOR controls translation of synaptic proteins, thereby critically sustaining neurocognitive performance. Dysregulation of this pathway has been implicated in several neuropsychiatric disorders, including Alzheimer disease and schizophrenia^37,40^. Although placental mTOR suppression has been extensively characterized as a mediator of impaired fetal growth in FGR^41^, it remains unclear whether brain mTOR signaling responds to metabolic perturbations and contributes to cognitive dysfunction.

Here, our study reveals downregulation of SIRT6 in hippocampal excitatory neurons of adult FGR mice. Through CaMKIIα-Cre-mediated neuronal SIRT6 conditional knockout (cKO), we establish that SIRT6 deficiency impairs synaptic plasticity and cognition by upregulating the TDO2/KYNA axis and suppressing mTOR signaling. Notably, SIRT6 overexpression normalized KYNA production, rescued mTOR pathway activity, and reversed synaptic deficits and cognitive impairments in FGR mice.

## Materials and methods

### Animals

Adult C57BL/6J mice were obtained from Shanghai SLAC Laboratory Animal Company and housed under specific pathogen-free conditions with controlled temperature, humidity, and a 12-h light-dark cycle. Females were mated with males, and the presence of a vaginal plug the next morning was defined as embryonic day 0.5 (E0.5). To establish the FGR model, pregnant mice at E0.5 were randomly assigned to either a control diet (20% protein) or a low-protein diet (8% protein) until delivery, after which all dams were fed the control diet. The offspring were raised under standard conditions and used for subsequent behavioral experiments. *Sirt6* flox/flox mice (C57BL/6J background) were kindly provided by Z. Mao (Tongji University), and CaMKIIα-Cre mice (C57BL/6J background) were purchased from Shanghai Model Organisms Center. Genotyping of offspring was performed using tail DNA and PCR with primers listed in Supplementary Table 1. In behavioral experiments, each group consisted of mice derived from three to five litters. All animal procedures were approved by the Institutional Animal Care and Use Committee of Tongji University (TJAB04520101).

### Novel object recognition test and novel location recognition test

Mice were habituated for 3 days in a black acrylic arena (25 × 25 × 25 cm), exploring two identical objects placed diagonally for 15 min/day. On day 4, one object was replaced with a novel object of identical size, and mice were allowed to explore for 6 min. Two days later, the familiar object was moved to a new location for novel location recognition (NLR). The first minute was considered acclimation; exploration during the subsequent 5 min was quantified. Exploration was defined as the mouse sniffing or touching the object with its nose/mouth within a 2 cm radius. For novel object recognition (NOR): discrimination index = (time exploring novel object – time exploring familiar object)/total exploration time × 100; discrimination ratio = time exploring novel object/total exploration time. For NLR: discrimination index = (time exploring relocated object – time exploring stationary object)/total exploration time × 100; discrimination ratio = time exploring relocated object/total exploration time.

### Morris water maze test

Before behavioral testing, mice underwent a 7-day handling protocol (5 min/day) to minimize experimenter-induced stress. Spatial navigation training was conducted in a Morris water maze paradigm using a white polypropylene circular pool (120 cm diameter) filled with opacified water (22 ± 1 °C). The pool environment featured distal spatial cues and was virtually divided into four quadrants, with a transparent acrylic escape platform (10 cm diameter) submerged 1.5 cm below water surface in the target quadrant. Spatial acquisition training occurred over 5 consecutive days with four trials per day (15 min intertrial interval). Start positions were pseudorandomized across four cardinal directions (T, O, L, R) to prevent procedural learning bias. For unsuccessful trials (failure to locate platform within 60 s), mice were gently guided to the target platform and permitted 20 s of spatial encoding. On day 6, the platform was removed for a 60-s probe trial, and behavior was recorded using the JLBehv-MWMM tracking system (Shanghai JiLiang Software Technology).

### Stereotaxic injection

Mice were anesthetized via intraperitoneal (i.p.) injection of 2.5% avertin (20 µl/g; Aladdin, A103416) and securely positioned in a stereotaxic frame (Stoelting, 51500). The surgical site was shaved, followed by disinfection and a midline scalp incision. The meninges were gently treated with 3% hydrogen peroxide (Aladdin, H299581) to enhance skull transparency, after which the bregma was identified as the stereotaxic reference point. Recombinant adeno-associated viruses (AAV2/9), designed and packaged by Shanghai OBiO Technology (Shanghai, China), were utilized for hippocampal targeting. Viral preparations had a validated titer of 1.0 × 10^12^ genome copies per milliliter (GC/ml). Before injection, AAVs were diluted 1:3 in sterile PBS, yielding a final volume of 2 μl per hemisphere. Bilateral microinjections into the CA1 subregion of the dorsal hippocampus were performed using the following stereotaxic coordinates relative to bregma: anteroposterior: −1.8 mm; mediolateral: ±1.4 mm; dorsoventral: −1.5 mm. Viruses were infused through a 33-gauge Hamilton syringe (5 μl capacity) at a rate of 0.2 μl/min. To minimize backflow, the injection needle remained in place for 10 min post-infusion before gradual withdrawal. Mice were housed individually and monitored during the recovery period. Behavioral experiments were initiated 4 weeks after viral delivery to ensure optimal transgene expression.

### Intragastric administration of TDO2 inhibitor

The TDO2 inhibitor 680C91 (Selleck, S8997) was initially dissolved in dimethyl sulfoxide (DMSO) to generate a 100 mM stock solution. Immediately before administration, the stock solution was diluted in sterile distilled water to achieve a working concentration of 1 μg/μl ^42^. A matched vehicle control containing equivalent concentrations of DMSO and water was prepared in parallel. Administration was performed daily via intragastric gavage (i.g.) using a 1.5-inch stainless steel 18-gauge feeding needle. Mice received 7.5 mg/kg body weight of 680C91 each morning, with treatments administered 6 days per week over a 6-week period.

### Intraperitoneal injection of MHY1485

MHY1485 (Selleck, S7811) was dissolved in DMSO to a 25.8 mM stock solution and then diluted in a vehicle composed of 10% DMSO, 40% PEG300, 5% Tween-80, and 45% saline to achieve a working concentration of 1 μg/μl. On the basis of previously published protocols with minor modifications, mice received i.p. injections at a dosage of 10 mg/kg/day for 4 consecutive weeks^43^.

### Golgi staining

Golgi staining was performed according to the manufacturer’s protocol using the FD Rapid GolgiStain Kit (FD Neuro Technologies, PK401). Coronal brain sections (100-μm thick) were examined under an OLYMPUS BX53 bright-field microscope. Neuronal dendritic arborization was quantitatively evaluated using the NeuroJ plugin in ImageJ software (NIH), with a minimum of four neurons analyzed per animal in each experimental group. Dendritic complexity was quantified by measuring the number of dendritic branches and total dendritic length. Additionally, Sholl analysis was conducted using ImageJ to further characterize dendritic branching patterns. For spine density analysis, images were acquired using a 100× oil-immersion objective, with results expressed as the mean number of spines per micrometer of dendrite length per neuron. Both spine density and morphological classification were based on previously published protocols^44^. On the basis of the length and width of dendritic spines, they can be categorized as: (1) filopodia (>2 μm in length); (2) long thin spines (<2 μm in length); (3) thin spines (<1 μm in length); (4) stubby spines (length-to-width ratio <1); (5) mushroom spines (>0.6 μm in head width); and (6) branched, with two or more heads.

### Long-term potentiation

Mice were deeply anesthetized and transcardially perfused with oxygenated ice-cold artificial cerebrospinal fluid (ACSF). The brain was rapidly dissected, and 400 μm hippocampal slices were prepared using a vibratome (Leica, 1000S). Slices were recovered in oxygenated ACSF at room temperature for at least 1 h. Field excitatory postsynaptic potentials (fEPSPs) were recorded in the CA1 stratum radiatum using ACSF-filled glass electrodes. Schaffer collateral fibers in CA3 were stimulated using a concentric bipolar electrode. Input–output curves were determined, and baseline fEPSPs were recorded for 10 min at 50–60% of maximal response. Long-term potentiation was induced by high-frequency stimulation (three 100 Hz trains of 100 pulses each, 0.1 ms duration, 20 s interval). Post-tetanic fEPSPs were recorded for 60 min and analyzed using the PATCHMASTER software (HEKA Electronics).

### RNA extraction, reverse transcription, and quantitative PCR

Total RNA from hippocampal tissues or cultured cells was extracted using RNAiso Plus (Takara, 108-95-2), and 500 ng RNA was reverse transcribed using the PrimeScript RT reagent kit (TaKaRa, RR037A). Quantitative PCR (qPCR) was performed on an Agilent Stratagene Mx3000P using SYBR Green Master Mix (Bio-Rad, 1725125). Primer sequences are listed in Supplementary Table 1. Relative gene expression was calculated using the 2^−ΔΔct^ method, with results normalized to Actin.

### Immunofluorescence

For cultured neurons, cells were fixed with 4% paraformaldehyde (PFA) for 20 min, permeabilized with 0.2% Triton X-100/PBS for 8 min, and blocked with 10% donkey serum/PBS for 1 h at room temperature. Primary antibodies (diluted in 10% donkey serum/PBS) were incubated overnight at 4 °C. For brain sections, mice were anesthetized and perfused transcardially with PBS followed by 4% PFA. Brains were post-fixed in 4% PFA for 24 h, and cryoprotected by 20% sucrose/PBS for 24 h, followed by 30% sucrose/PBS twice every 24 h. Coronal sections (35 μm) were prepared using a freezing microtome (Leica CM3050 S, Germany) and stored in brain freezing medium. After heat retrieval, sections were blocked with PBS containing 10% donkey serum and 0.2% Triton X-100 for 1 h at room temperature and then incubated overnight with primary antibodies at 4 °C (diluted in PBS with 5% donkey serum and 0.1% Triton X-100). Both cells and brain sections were incubated in secondary antibodies and Hoechst for 1 h at room temperature in the dark. Images were acquired using a Leica TCS SP8 confocal microscope. *Z*-stack projections were obtained for tissue samples. Antibody details are listed in Supplementary Table 2.

### Western blot

Samples were lysed in RIPA buffer (Beyotime, P0013B) supplemented with protease inhibitors (Roche, 04693132001) and phosphatase inhibitors (Roche, 4906845001). Protein concentrations were quantified using a BCA assay (Vazyme, E112-01). Equal amounts of protein were resolved on SDS–PAGE gels, transferred to polyvinylidene fluoride membranes (PerkinElmer), and blocked in 3% bovine serum albumin in TBST (Tris-buffered saline, 0.1% Tween-20) for 1 h. Membranes underwent overnight incubation with primary antibodies at 4 °C, followed by three 5-min TBST washes. Subsequently, membranes were probed with species-matched horseradish peroxidase-conjugated secondary antibodies for 1 h at temperature, with three additional TBST washes (5 min each) performed post-incubation. Signals were detected using the Enhanced ECL Chemiluminescent Substrate Kit (Yeasen, 36222) and visualized by an imaging system (ImageQuant LAS 4000). The membrane was stained with Ponceau S (Solarbio, P0012) for 10 min. Band intensity was quantified using ImageJ. Antibody information, full blot images, and quantification data were presented in Supplementary Table 2 and Source Data file.

### Chromatin immunoprecipitation-qPCR

The methods and solution preparations were adapted with minor modifications from previously published protocols^11^. Hippocampal CA1 tissues (one mouse per sample) were homogenized in 450 μl PBS through sequential 18G/21G needle extrusion, followed by 10-min formaldehyde crosslinking, quenching with 1.25 M glycine, and triple ice-cold PBS washes. After centrifugation (1,500 rpm, 4 °C, 10 min), pellets were resuspended in Cell Lysis Buffer for 15 min ice incubation, followed by nuclear isolation (3,000 rpm, 5 min). Nuclear pellets underwent lysis in 400 μl Nuclei Lysis Buffer and chromatin fragmentation using a Covaris M220 ultrasonicator (500–1,000 bp fragments; parameters: 75 W peak power, 10% duty factor, 150 cycles per burst, 10 min at 4 °C). Cleared lysates were immunoprecipitated overnight at 4 °C with either IgG control or target-specific antibodies (Supplementary Table 2), followed by 2-h capture with Protein G Magnetic Beads (20 μl, 9006S, Cell Signaling Technology). After sequential buffer washes, complexes were eluted in chromatin immunoprecipitation elution buffer, reverse-crosslinked (65 °C, overnight), and treated with RNase A (0.2 μg/ml, 37 °C for 1 h) and Proteinase K (0.2 μg/ml, 55 °C for 1 h). DNA purification used phenol–chloroform extraction (Solarbio, P1012) and ethanol precipitation. Input controls used 10% nuclear extract DNA. According to refs. ^45,46^, the promoter region of the known SIRT6 target gene RPL23 served as the positive control, whereas an intergenic region predicted to lack SIRT6 binding served as the negative control. Target enrichment was quantified by qPCR (primers: Supplementary Table 1) and expressed as fold change over IgG controls. Antibody information was presented in Supplementary Table 2.

### RNA-sequencing data analysis

Total RNA was extracted from the hippocampus of individual 8-week-old male mice using the RNAiso Plus (Takara, 108-95-2). RNA quality was assessed using the Agilent Bioanalyzer 2100. Library construction and sequencing were performed by Novogene (Beijing, China). Adapter trimming was performed using Trim Galore (v0.6.5), and reads were aligned to the mouse genome (mm10) using Bowtie2 (v2.4.5). Duplicates were marked with SAMtools (v1.3.1), and gene counts and transcripts per million were calculated using HTSeq (v2.0.3). Genes with total counts <10 were excluded. Differential expression analysis was performed using DESeq2 (v1.32.0), with Benjamini–Hochberg correction. Gene Ontology (GO) and KEGG pathway analysis were performed via DAVID, and heatmaps were generated with pheatmap (v1.0.12) in R (v4.1.3). Gene set enrichment analysis (GSEA) was conducted using ClusterProfiler (v4.10.1).

### Statistical analysis

Statistical analysis was performed using GraphPad Prism 10.0. Data are presented as mean ± SEM from at least three independent experiments. Normality was assessed using the Shapiro–Wilk test or the Kolmogorov–Smirnov test. Homogeneity of variance was tested using *F*-test (for unpaired *t* tests), Brown–Forsythe test (for one-way analysis of variance (ANOVA)), or Levene’s test (for two-way ANOVA). Statistical significance was determined using unpaired two-tailed Student’s *t* tests (for two-group comparisons), one-way ANOVA followed by Tukey’s test (for multiple group comparisons), or two-way ANOVA with Sidak’s post hoc test (for multifactorial designs). Given the litter effect, animal behavioral experiments were analyzed using a linear mixed-effects model (IBM SPSS Statistics v27.0; SPSS Inc., USA), with genotype or treatment group as the fixed effect and litter as the random effect^47^. Significance thresholds were set at *P < 0.05, **P < 0.01, and ***P < 0.001. Detailed statistical data for all figures are available in the Source Data file.

## Results

### FGR decreases SIRT6 in hippocampal glutamatergic neurons

**Fig. 1:**
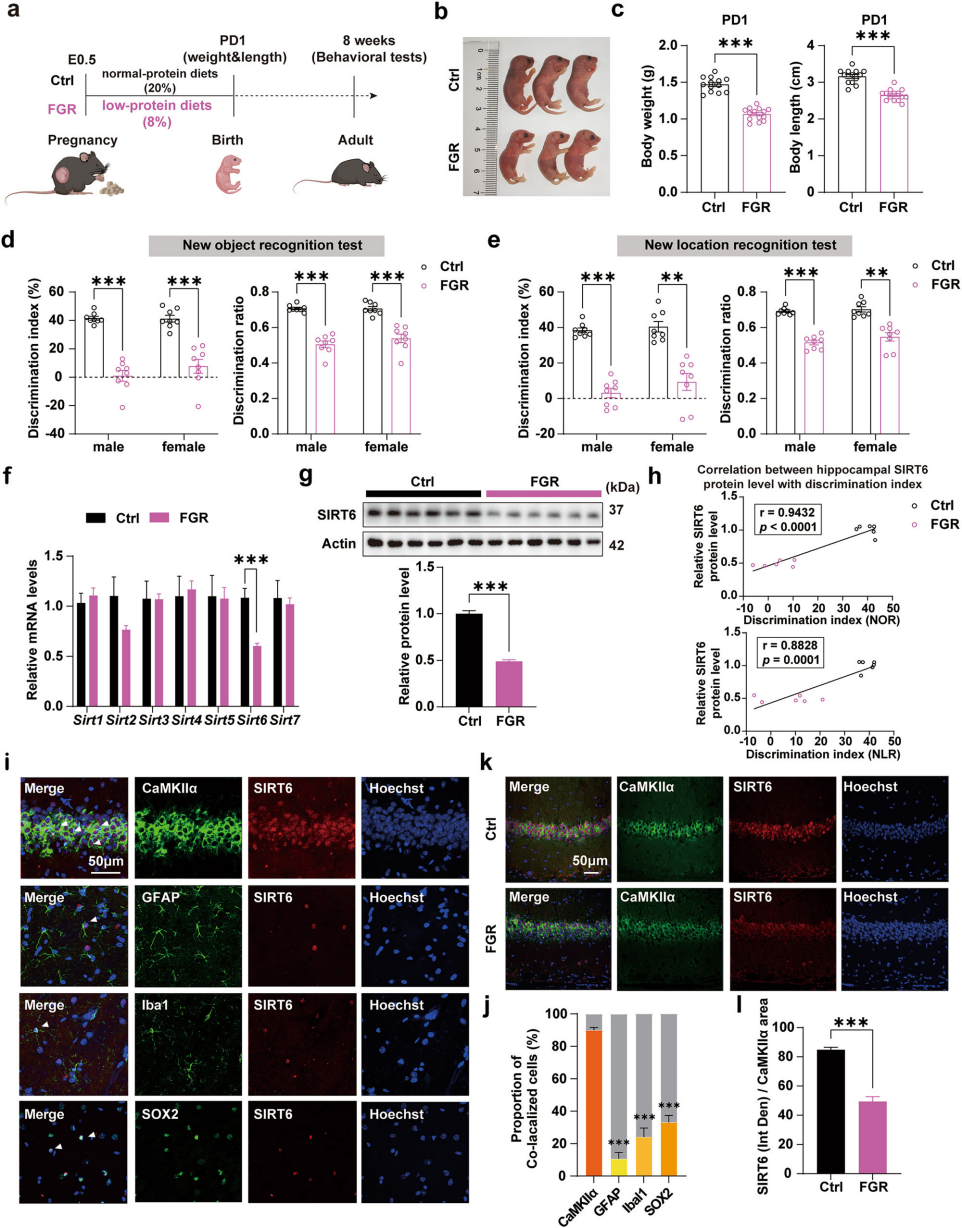
FGR leads to reduced SIRT6 expression in hippocampal glutamatergic neurons. **a** Schematic timeline of the fetal growth restriction (FGR) mouse model. **b** Representative photographs of Ctrl and FGR mice at PD1. **c** Average body weight and body length of Ctrl and FGR mice at PD1 (Ctrl, *n* = 13/3 litters; FGR, *n* = 14/4 litters). **d** Discrimination index and discrimination ratio of the novel object recognition (NOR) test in Ctrl and FGR mice (*n* = 8 mice/5 litters per group). **e** Discrimination index and discrimination ratio of the novel location recognition test in Ctrl and FGR mice (*n* = 8 mice/5 litters per group). **f** Quantitative PCR analysis of *Sirt1-7* mRNA levels in the hippocampus of Ctrl and FGR mice (*n* = 6 mice per group). **g** Western blot analysis of SIRT6 protein level in the hippocampus of Ctrl and FGR mice (*n* = 6 mice per group). **h** Positive correlation of SIRT6 protein level in the hippocampus with discrimination index in NOR and NLR tests (*n* = 6 mice per group). **i** Representative images showing SIRT6 expression in CaMKIIα^+^, GFAP^+^, Iba1^+^, and SOX2^+^ cells. Scale bar, 50 μm. **j** Quantification of percentage of SIRT6^+^ cells immunoreactive to CaMKIIα, GFAP, Ibal1, or SOX2. Representative images (part **k**) and quantification of SIRT6 (part **l**) in hippocampal CA1 neurons of Ctrl and FGR mice (*n* = 3 mice per group). Statistics are derived from eight slices. Scale bar, 50 μm. The data are shown as mean ± SEM. Two-tailed Student’s *t* test in parts **c**, **f**, **g**, and **l**; one-way analysis of variance with Tukey’s multiple comparisons test in part **j**; linear mixed-effects model in parts **d** and **e**. ***P < 0.001.

Using a validated prenatal low-protein diet model (Fig. 1a), we established FGR as demonstrated by significantly lower body weight and body length in neonatal mice at postnatal day 1 (PD1) (Fig. 1b,c). Behavioral phenotyping at adulthood (postnatal 8 weeks) revealed deficits in recognition memory, with both the discrimination index and ratio significantly decreased in the NOR and NLR tests across sexes (Fig. 1d,e). qPCR analysis identified a 44.36% reduction in hippocampal Sirt6 mRNA without alterations in other sirtuin isoforms (Fig. 1f). Consistent with transcriptional changes, western blot (WB) analysis confirmed a 50.98% decrease in SIRT6 protein expression (Fig. 1g). Strikingly, hippocampal SIRT6 levels showed robust positive correlations with cognitive performance (NOR: *r* = 0.9432, *P* < 0.0001; NLR: *r* = 0.8828, *P* = 0.0001) (Fig. 1h). Cellular specificity analysis revealed predominant SIRT6 localization to CaMKIIα^+^ excitatory neurons (co-expression: 89.93 ± 0.77%), with minimal expression in glial populations (Iba1^+^ microglia: 24.11 ± 2.48%; GFAP^+^ astrocytes: 10.65 ± 1.78%) and neural precursors (SOX2^+^ cells: 33.16 ± 1.88%) (Fig. 1i,j). Quantitative immunofluorescence (IF) demonstrated a marked reduction in SIRT6 intensity within CaMKIIα^+^ neurons of FGR mice (Fig. 1k,l). These findings collectively demonstrate that FGR-induced cognitive deficits closely correlate with glutamatergic neuron-specific SIRT6 reduction.

### Excitatory neuron-specific SIRT6 deletion impairs hippocampus-dependent cognition

**Fig. 2:**
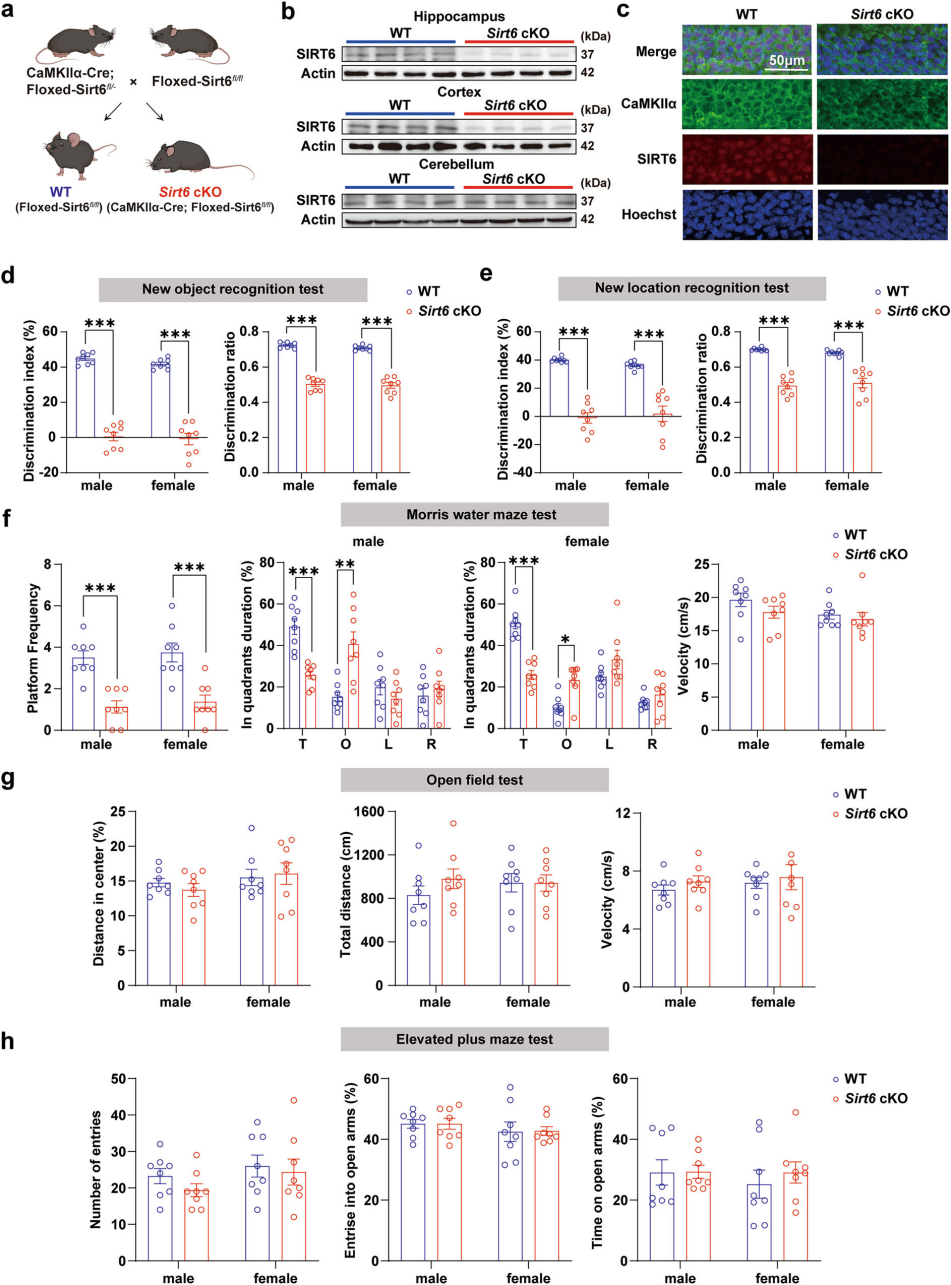
*Sirt6* cKO mice display learning and memory deficits. **a** Breeding scheme of *Sirt6* cKO and littermate control (wild-type (WT)) mice. **b** Representative western bolt images of SIRT6 protein levels in the hippocampus, cortex, and cerebellum of WT and *Sirt6* cKO mice (*n* = 4 mice per group). **c** CaMKIIα^+^ hippocampal neurons in WT mice express SIRT6, whereas SIRT6 is absent from these neurons in *Sirt6* cKO mice, confirming SIRT6 deletion in excitatory neurons. Scale bar, 50 μm. **d** Discrimination index and discrimination ratio of the novel object recognition test in WT and *Sirt6* cKO mice (*n* = 8 mice/5 litters per group). **e** Discrimination index and discrimination ratio of the novel location recognition test in WT and *Sirt6* cKO mice (*n* = 8 mice/5 litters per group). **f** Morris water maze performance in WT and *Sirt6* cKO mice. Platform frequency, percentage of time spent in the target quadrant, and swim velocity are shown (*n* = 8 mice/5 litters per group). **g** Open field test results for WT and *Sirt6* cKO mice (*n* = 8 mice/5 litters per group). **h** Elevated plus maze test results for WT and *Sirt6* cKO mice (*n* = 8 mice/5 litters per group). The data are shown as mean ± SEM. Linear mixed-effects model in parts **d**–**h**. **P < 0.01 and ***P < 0.001.

To investigate the causal role of glutamatergic SIRT6 in cognitive function, we generated cKO mice by crossing *Sirt6* floxed strains with CaMKIIα-Cre transgenics^48^ (Fig. 2a). Genomic PCR confirmed efficient recombination of the floxed allele (Supplementary Fig. 1a). Regional qPCR analysis revealed a 68.3% reduction in hippocampal *Sirt6* mRNA and a 54.7% decrease in cortical expression, with cerebellar levels unchanged (Supplementary Fig. 1b), consistent with CaMKIIα promoter specificity. Immunoblotting validated a 66.0% reduction in hippocampal SIRT6 protein and 68.4% decrease in cortex, whereas cerebellar expression remained intact (Fig. 2b and Supplementary Fig. 1c). IF further demonstrated SIRT6 loss in CaMKIIα^+^ hippocampal neurons (Fig. 2c), confirming cell-type-specific KO efficacy.

Behavioral tests were conducted to assess hippocampus-dependent cognitive functions in adult *Sirt6* cKO mice. In both the NOR and NLR tasks, *Sirt6* cKO mice exhibited significant reductions in both the discrimination index and ratio versus WT littermates (Fig. 2d,e). Spatial memory impairments were evident in the Morris water maze test, with cKO mice exhibiting fewer platform crossings and less time in the target quadrant, despite comparable swim speed (Fig. 2f). Anxiety tests^49^ ruled out locomotor or emotional bias: no difference in central zone exploration, total distance, or velocity in the open field test (Fig. 2g), nor in the number, percentage, or duration of open arm entries in the elevated plus maze test (Fig. 2h). Sex-independent phenotypes strengthen the causal link between glutamatergic SIRT6 loss and cognitive decline. Taken together, these findings demonstrate that the deletion of Sirt6 in excitatory neurons leads to hippocampus-dependent learning and memory deficits in adult mice.

### Loss of SIRT6 impairs hippocampal synaptic plasticity

**Fig. 3:**
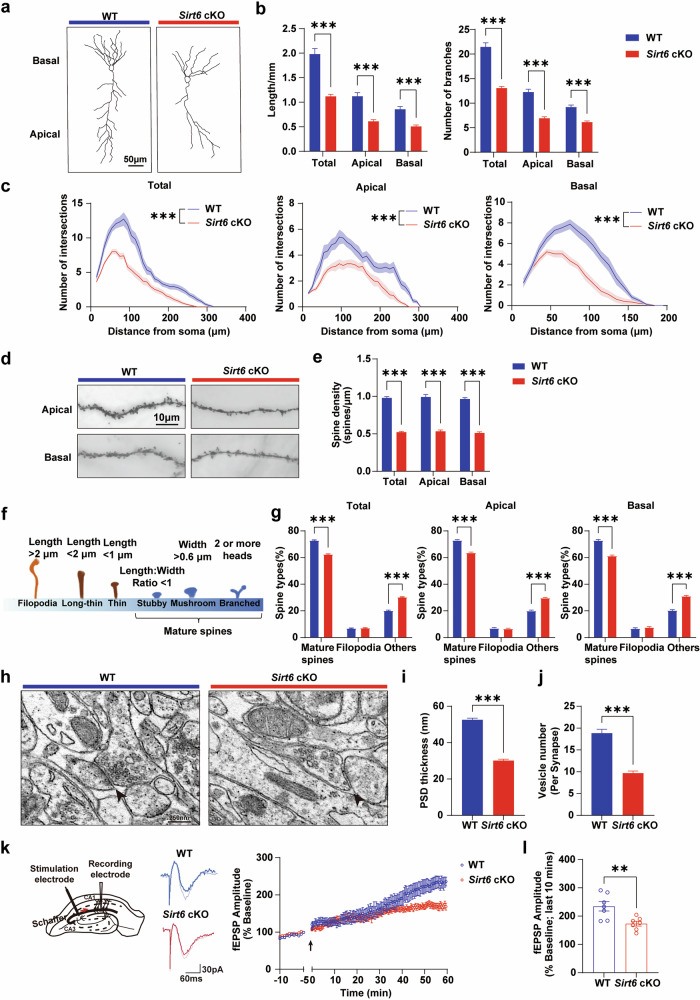
*Sirt6* cKO mice exhibit impaired hippocampal synaptic plasticity. **a** Representative Golgi staining images of hippocampal CA1 neurons from wild-type (WT) and *Sirt6* conditional knockout (cKO) mice. Scale bar, 50 μm. **b** *Sirt6* cKO mice show reduced dendritic length and fewer dendrites compared with WT mice (*n* = 15 neurons from 3 mice per group). **c** Sholl analysis of dendritic complexity in neurons from the WT and *Sirt6* cKO groups (*n* = 15 neurons from 3 mice per group). Representative spine images (part **d**) and quantitative analysis of spine density (part **e**) in CA1 neurons from WT and *Sirt6* cKO mice (*n* = 15 neurons from 3 mice per group). Scale bar, 10 μm. **f** Schematic diagram of different morphologies of dendritic spines. **g**, Quantitative analysis of the percentage of spine types in WT and *Sirt6* cKO mice (*n* = 15 neurons from 3 mice per group). **h** Representative transmission electron microscopy images of synaptic contacts in CA1 neurons from WT and *Sirt6* cKO mice. The structure indicated by the arrow is a synapse. Scale bar, 250 nm. Quantitative analysis of postsynaptic density (PSD) length (part **i**) and synaptic vesicle number (part **j**) in CA1 synapses of WT and *Sirt6* cKO mice (*n* = 60 synapses from 3 mice per group). Plots of normalized field excitatory postsynaptic potential (fEPSP) slopes recorded in CA1 over time before and after the induction of long-term potentiation (part **k**) and average long-term potentiation amplitude in acute slices from WT and *Sirt6* cKO mice (part **l**) (*n* = 7 slices from 3 mice per group). The data are shown as mean ± SEM. Two-tailed Student’s *t* test in parts **b**, **e**, **g**, **i**, **j**, and **l**; two-way analysis of variance followed by Sidak’s multiple comparisons test was used for multiple comparisons in part **c**. **P < 0.01 and ***P < 0.001.

To further investigate the role of SIRT6 in the structural and functional integrity of hippocampal excitatory neurons, we first analyzed CA1 pyramidal neuron morphology in *Sirt6* cKO mice. Using Golgi staining, *Sirt6* cKO neurons exhibited significantly reduced dendritic length and branch numbers in both apical and basal arbors versus WT (Fig. 3a,b). Sholl analysis confirmed diminished dendritic complexity in *Sirt6*-deficient neurons (Fig. 3c). Moreover, dendritic spine density was significantly decreased in both apical and basal dendrites of *Sirt6* cKO neurons (Fig. 3d,e), accompanied by a reduction in the proportion of mature spines, an unchanged percentage of filopodia, and an increase in other immature spine types (Fig. 3f,g). Transmission electron microscopy revealed ultrastructural abnormalities in CA1 synapses of *Sirt6* cKO mice (Fig. 3h), including thinner postsynaptic density (PSD) layers (Fig. 3i) and fewer synaptic vesicles (Fig. 3j). Electrophysiological recordings showed that although theta burst stimulation induced long-term potentiation in both groups, the amplitude of fEPSPs was significantly lower in cKO slices (Fig. 3k,l), indicating impaired synaptic plasticity. In addition, we cultured primary hippocampal neurons from PD1 pups and knocked down *Sirt6* using lentiviral short hairpin RNA (Supplementary Fig. 1d). SIRT6 knockdown significantly reduced total dendritic length, branching number, and complexity (Supplementary Fig. 1e–g). Synaptic density and the proportion of functional synapses (Synapsin1^+^PSD95^+^ puncta) were also markedly reduced (Supplementary Fig. 1h,i). These convergent in vivo and in vitro results demonstrate a crucial role for SIRT6 in maintaining dendritic morphogenesis and synaptic plasticity of excitatory hippocampal neurons.

### SIRT6 regulates synaptic plasticity and cognition via its deacetylase activity

**Fig. 4:**
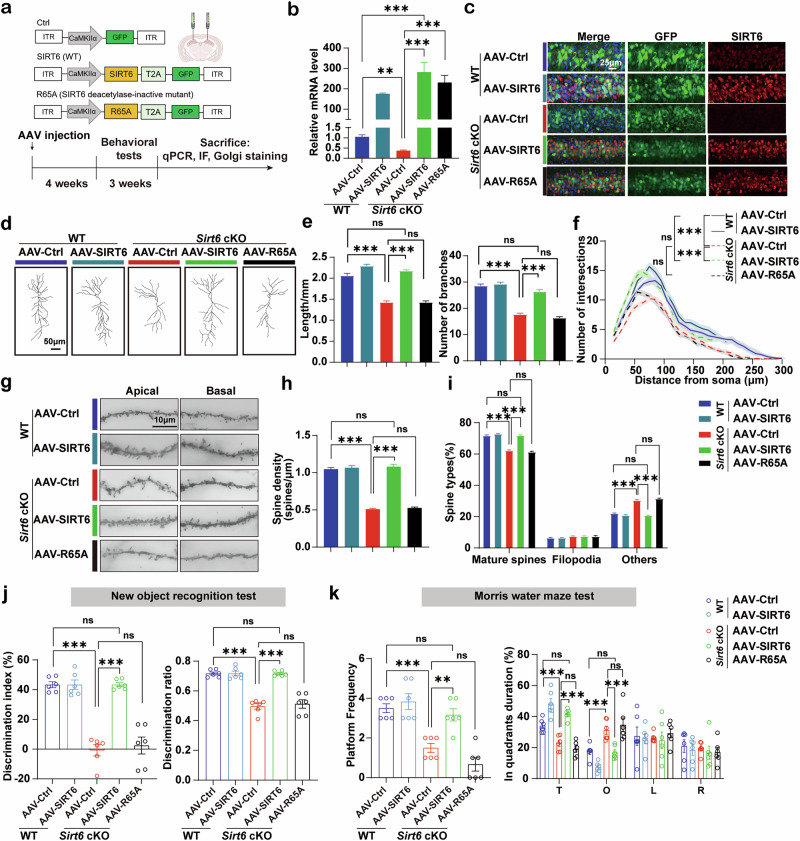
SIRT6 regulates synaptic plasticity and cognition depending on its deacetylase activity. **a** Schematic of the adeno-associated virus (AAV) construct expressing green fluorescent protein (GFP), wild-type SIRT6, or the R65A mutant under the control of the CaMKIIα promoter and experimental timeline. **b** Quantitative PCR (qPCR) analysis of Sirt6 mRNA level in the hippocampus following AAV injection from WT + AAV-Ctrl, WT + AAV-SIRT6, *Sirt6* cKO + AAV-Ctrl, *Sirt6* cKO + AAV-SIRT6, and *Sirt6* cKO + AAV-R65A groups (*n* = 3 mice per group). **c** Immunofluorescence (IF) showing SIRT6 (red) and GFP (green) in the CA1 region of five groups. Scale bar, 25 μm. **d** Representative Golgi staining images of the hippocampal CA1 neurons from the five groups. Scale bar, 50 μm. **e** Quantification of total dendritic length and branch number of hippocampal CA1 neurons from five groups (*n* = 15 neurons from 3 mice per group). **f** Sholl analysis of dendritic complexity in neurons from five groups (*n* = 15 neurons from 3 mice per group). **g** Representative Golgi staining images of dendritic spines in hippocampal CA1 neurons from five groups. Scale bar, 10 μm. Quantitative analysis of spine density (part **h**) and the percentage of spine types (part **i**) (*n* = 15 neurons from 3 mice per group). **j** Discrimination index and discrimination ratio of the novel object recognition test across the five groups (*n* = 6 mice/5 litters per group). **k** Platform frequency, the percentage of duration in four quadrants during the Morris water maze test in the five groups (*n* = 6 mice/5 litters per group). The data are shown as mean ± SEM. Two-tailed Student's *t* test in part **b**, One-way analysis of variance with Tukey’s multiple comparisons test in parts **e**, **h**, and **i**; two-way analysis of variance followed by Sidak’s multiple comparisons test was used for multiple comparisons in part **f**; linear mixed-effects model in parts **j** and **k**. **P < 0.01 and ***P < 0.001. cKO, conditional knockout; ns, not significant; WT, wild-type.

To dissect the enzymatic basis of neuroprotective role of SIRT6, we engineered four catalytic mutants. The G60A mutant retains only deacetylase activity, whereas the R65A mutant maintains mono-ADP-ribosyltransferase activity. The S56Y and H133Y variants are catalytically inactive^50^. WB analysis confirmed comparable protein expression levels of all mutants. Only G60A preserved deacetylase activity, as evidenced by its ability to reduce H3K9 acetylation (H3K9ac) compared with SIRT6 WT, whereas R65A, S56Y, and H133Y mutants showed no activity, validating successful construction of enzymatically distinct mutants (Supplementary Fig. 2a). In SIRT6-knockdown neurons, only SIRT6 WT and G60A restored dendritic length, branch numbers and complexity (Supplementary Fig. 2b–e), synaptic density, and the proportion of functional synapses (Supplementary Fig. 2f,g), whereas R65A, S56Y, and H133Y mutants showed no rescue, indicating that deacetylase — but not mono-ADP-ribosylation — activity is essential for dendritic development and synaptogenesis. For in vivo validation, we used CaMKIIα-driven AAV vectors to selectively overexpress SIRT6 WT or R65A in hippocampal excitatory neurons of *Sirt6* cKO mice (Fig. 4a–c). Golgi staining showed that overexpression of SIRT6 fully rescued dendritic length, branch numbers, and complexity (Fig. 4d–f and Supplementary Fig. 2h–j), as well as spine density (Fig. 4g,h and Supplementary Fig. 2k) and the proportion of mature spines (Fig. 4i and Supplementary Fig. 2l). By contrast, R65A failed to produce these effects. Behaviorally, SIRT6, but not R65A, restored the discrimination index and ratio in the NOR test (Fig. 4j). Similarly, SIRT6 improved platform crossing and target quadrant dwell time in the Morris water maze test, whereas R65A showed no benefit (Fig. 4k). Notably, SIRT6 overexpression in WT mice did not further enhance dendritic development, synaptic density, or cognitive performance, suggesting that SIRT6 functions in a physiologically constrained manner. These results collectively demonstrate that the deacetylase activity of SIRT6, rather than its ADP-ribosyltransferase function, is critical for maintaining dendritic architecture, synaptic plasticity, and cognitive ability in hippocampal excitatory neurons.

### TDO2 is a key transcriptional target of SIRT6-H3K9 deacetylation in regulating synaptic and cognitive function

**Fig. 5:**
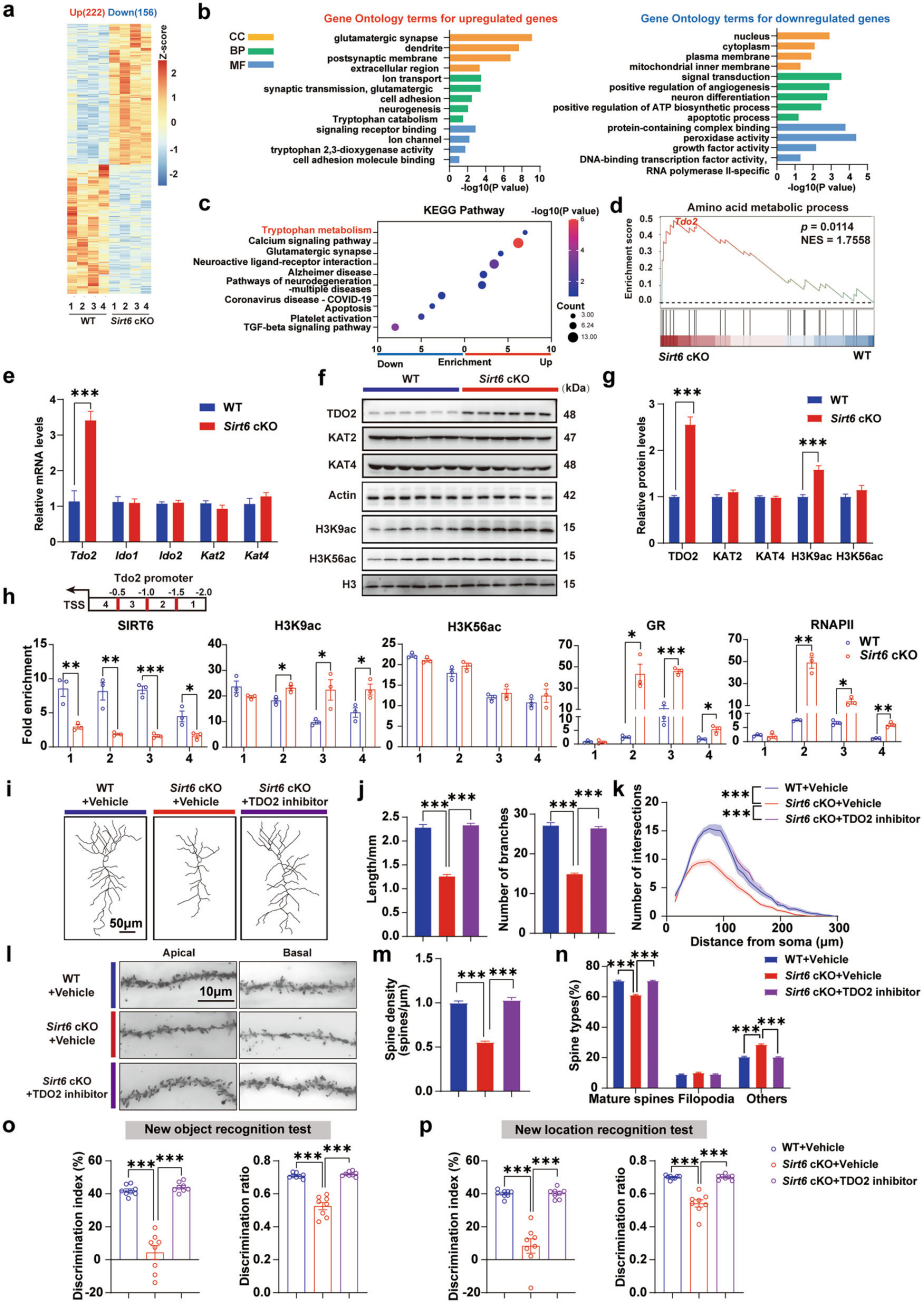
TDO2 is a key downstream effector of SIRT6-mediated H3K9 deacetylation, driving synaptic and cognitive deficits rescued by TDO2 inhibition. **a** Heat map of differentially expressed genes in the hippocampus of *Sirt6* conditional knockout (cKO) and wild-type (WT) mice. **b** Gene Ontology enrichment analysis of upregulated and downregulated genes in *Sirt6* cKO mice, categorized into cellular component (CC), biological process (BP), and molecular function (MF). **c** KEGG pathway enrichment analysis of differentially expressed genes in the hippocampus of *Sirt6* cKO mice. **d** Gene set enrichment analysis results of positive enrichment of amino acid metabolic process in the hippocampus of *Sirt6* cKO mice. NES: Normalized Enrichment Score. **e** Quantitative PCR analysis of *Tdo2*, *Ido1*, *Ido2, Kat2*, and *Kat4* mRNA levels in the hippocampus of WT and *Sirt6* cKO mice (*n* = 5 mice per group). Representative western blot images (part **f**) and quantitative PCR analysis (part **g**) of TDO2, KAT2, KAT4, H3K9ac, and H3K56ac protein levels in the hippocampus from WT and *Sirt6* cKO mice (*n* = 6 mice per group). **h** Chromatin immunoprecipitation-quantitative PCR analysis showing the enrichment of SIRT6, H3K9ac, H3K56ac glucocorticoid receptor, and RNA polymerase II (RNAPII) at four promoter regions of *Tdo2* (*n* = 3 mice per group). **i** Representative Golgi staining images of hippocampal CA1 neurons from WT + Vehicle, *Sirt6* cKO + Vehicle, and *Sirt6* cKO + TDO2 inhibitor groups. Scale bar, 50 μm. Quantification of total dendritic length and branch number (part **j**) and Sholl analysis of dendritic complexity (part **k**) in the CA1 neurons across the three groups (*n* = 15 neurons from 3 mice per group). **l**, Representative Golgi staining images of dendritic spines in hippocampal CA1 neurons. Scale bar, 10 μm. Quantitative analysis of spine density (part **m**) and the percentage of spine types (part **n**) (*n* = 15 neurons from 3 mice per group). Discrimination index and discrimination ratio of the novel object recognition test (part **o**) and novel location recognition test (part **p**) across the three groups (*n* = 8 mice per group). The data are shown as mean ± SEM. Two-tailed Student’s *t* test in parts **e**, **g**, and **h**; one-way analysis of variance with Tukey’s multiple comparisons test in parts **j**, **m**, and **n**; two-way analysis of variance followed by Sidak’s multiple comparisons test was used for multiple comparisons in part **k**; linear mixed-effects model in part **p**. TGF-β, transforming growth factor-beta; TSS, transcription start site. *P < 0.05, **P < 0.01, and ***P < 0.001.

To identify the downstream targets of SIRT6, we isolated the hippocampi of adult *Sirt6* cKO mice and their littermates and performed RNA-sequencing. A cut-off of false discovery rate < 0.05 and |fold change | ≥ 1.5 was used to identify differentially expressed genes. SIRT6 deficiency caused 222 upregulated and 156 downregulated genes (Fig. 5a). GO enrichment analysis showed that upregulated genes were primarily localized to glutamatergic synapses, dendrites, and postsynaptic membranes and enriched for ion transport, synaptic transmission, cell adhesion, and TRP catabolism. By contrast, downregulated genes were enriched in nuclear, plasma membrane, and mitochondrial compartments and involved in signal transduction, angiogenesis, and ATP biosynthesis (Fig. 5b). KEGG pathway analysis revealed that upregulated genes were significantly enriched in TRP metabolism, calcium signaling, and neuroactive ligand–receptor interaction, whereas downregulated genes were associated with transforming growth factor-β signaling (Fig. 5c). GSEA revealed significant enrichment in amino acid metabolic pathways, with the TRP catabolism gene *Tdo2* achieving the highest enrichment score (*P* = 0.0114, NES = 1.7558) (Fig. 5d). qPCR analysis confirmed a significant upregulation of *Tdo2* mRNA in the hippocampus of *Sirt6* cKO mice (Fig. 5e). Immunoblotting showed the upregulation of TDO2 protein and H3K9ac in *Sirt6* cKO hippocampi, but no alteration in H3K56ac (Fig. 5f,g), suggesting that SIRT6 acts as a principal deacetylase for H3K9ac in hippocampal excitatory neurons. Notably, the expression of other TRP-KP enzymes, including Ido1, Ido2, and the primary KYNA-synthesizing enzymes in mice (KAT2 and KAT4)^51,52^, was unaffected (Fig. 5e–g). This specific upregulation of Tdo2 implicates a precise regulatory mechanism governed by SIRT6.

To elucidate SIRT6 regulation of *Tdo2* transcription, we identified glucocorticoid receptor (GR) binding sites within the mouse *Tdo2* core promoter (2 kb upstream of TSS) using JASPAR analysis (Supplementary Fig. 3a), consistent with reported GR binding sites in the human *TDO2* promoter^53^. In HT22 hippocampal neuronal cells, SIRT6 knockdown upregulated *Tdo2* expression; this effect was abolished by concurrent GR knockdown, demonstrating the essential role of GR as a mediator (Supplementary Fig. 3b,c). We then divided the *Tdo2* promoter into four contiguous 500-bp fragments and performed chromatin immunoprecipitation-qPCR analysis on hippocampal tissue from *Sirt6* cKO mice. SIRT6 deficiency specifically enhanced H3K9ac (but not H3K56ac), GR binding, and RNA polymerase II (RNAPII) recruitment in promoter regions 2–4, with no changes observed in distal region 1 (Fig. 5h and Supplementary Fig. 3d). These findings indicate that SIRT6 deficiency enhances Tdo2 transcription, likely by increasing H3K9ac to promote GR binding and RNAPII recruitment.

Given the identification of TDO2 as a transcriptional target downstream of SIRT6, we next sought to determine whether TDO2 mechanistically contributes to SIRT6-dependent synaptic impairments and cognitive deficits. We treated *Sirt6* cKO mice with the brain-penetrant TDO2 inhibitor 680C91 for 6 consecutive weeks^54^ (Supplementary Fig. 3e). Golgi staining revealed that 680C91 treatment restored dendritic length and branch numbers in *Sirt6* cKO CA1 neurons (Fig. 5i,j and Supplementary Fig. 3f), improved dendritic complexity (Fig. 5k and Supplementary Fig. 3g), and normalized spine density (Fig. 5l,m and Supplementary Fig. 3h) and the proportion of mature spines (Fig. 5n and Supplementary Fig. 3i). Behaviorally, 680C91-treated *Sirt6* cKO mice showed significant improvements in both NOR and NLR performance (Fig. 5o,p) and, in the Morris water maze test, exhibited restored platform crossings and time spent in the target quadrant, normalizing cognition to WT levels (Supplementary Fig. 3j). These data position TDO2 as the pivotal effector of SIRT6-mediated synaptic dysfunction and cognitive function.

### SIRT6–TDO2–KYNA axis disrupts synaptic plasticity via mTOR pathway suppression

**Fig. 6:**
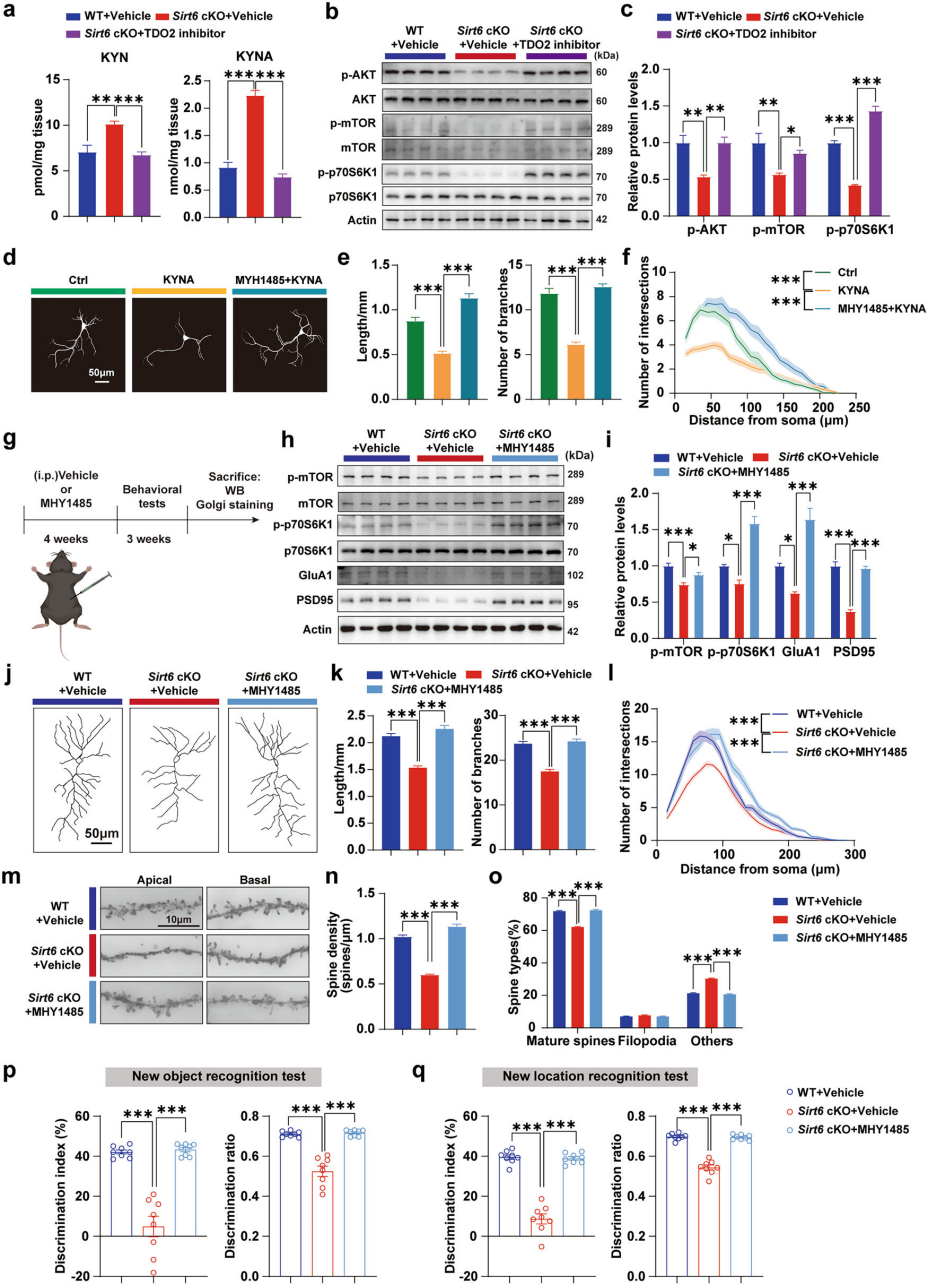
Activated TDO2/KYNA metabolic pathway impairs synaptic plasticity and cognition by downregulating mTOR signaling. **a** Enzyme-linked immunosorbent assay quantification of hippocampal kynurenine (KYN) and kynurenic acid (KYNA) levels in WT + Vehicle, *Sirt6* cKO + Vehicle, and *Sirt6* cKO + TDO2 inhibitor mice (*n* = 6 per group). Representative western blot (WB) images (part **b**) and quantification (part **c**) of p-AKT/AKT, p-mTOR/mTOR, and p-p70S6K1/p70S6K1 protein levels in the hippocampus from three groups (*n* = 4 mice per group). **d** Representative images of cultured hippocampal neurons immunostained for the neuronal marker MAP2 treated by DMSO (Ctrl), KYNA (1 mM), and MHY1485 (10 μM) + KYNA (1 mM). Scale bar, 50 μm. Quantification of total dendritic length and the branch number (part **e**) and Sholl analysis of dendritic morphology (part **f**) of primary hippocampal neurons from the four groups (*n* = 20 neurons from 6–8 mice). **g** Schematic of the experimental timeline for MHY1485 intraperitoneal (i.p.) administration (10 mg/kg). Representative WB images (part **h**) and quantification (part **i**) of p-mTOR/mTOR, p-p70S6K1/p70S6K1, GluA1, and PSD95 protein levels in the hippocampus from WT + Vehicle, *Sirt6* cKO + Vehicle, and *Sirt6* cKO + MHY1485 groups (*n* = 4 mice per group). **j**, Representative Golgi staining images of hippocampal CA1 neurons from three groups. Scale bar, 50 μm. **k** Quantification of total dendritic length and the branch number of hippocampal CA1 neurons (*n* = 15 neurons from 3 mice per group). **l** Sholl analysis of dendritic complexity in neurons from three groups (*n* = 15 neurons from 3 mice per group). **m** Representative images of dendritic spines in CA1 neurons. Scale bar, 10 μm. Quantification of spine density (part **n**) and the percentage of spine types (part **o**) (*n* = 15 neurons from 3 mice per group). Discrimination index and discrimination ratio of the novel object recognition test (part **p**) and novel location recognition test (part **q**) across the three groups (*n* = 8 mice/5 litters per group). The data are shown as mean ± SEM. One-way analysis of variance with Tukey’s multiple comparisons test in parts **a**, **c**, **e**, **i**, **k**, **n**, and **o**; two-way analysis of variance followed by Sidak’s multiple comparisons test was used for multiple comparisons in parts **f** and **l**; linear mixed-effects model in parts **p** and **q**. cKO, conditional knockout; PSD, postsynaptic density; WT, wild-type. *P < 0.05, **P < 0.01, and ***P < 0.001.

TRP is metabolized by TDO2 into KYN, a key precursor for the neuroactive metabolite KYNA^28,29^. *Sirt6* cKO hippocampi exhibited a significant increase in both KYN and KYNA levels, which were normalized by TDO2 inhibitor 680C91, confirming that TDO2 hyperactivity drives neurotoxic metabolite accumulation (Fig. 6a). Given the central role of the mTOR signaling cascade in metabolic sensing and protein synthesis critical for synaptic plasticity and cognition^37,38^, we investigated whether altered TRP metabolism mediates the effects of SIRT6 deficiency via suppression of the mTOR pathway. GSEA of *Sirt6* cKO transcriptomes revealed mTOR signaling as the significantly downregulated pathway (*P* = 0.0420, NES = 1.5232) (Supplementary Fig. 4a). Also, GO analysis of published *Sirt6* KO transcriptomes from monkeys^20^ showed mTOR pathway suppression (Supplementary Fig. 4b). Immunoblotting showed that Sirt6 deletion reduced phosphorylation of AKT (Ser473), mTOR (Ser2448), and p70S6K1 (Thr389), effects all rescued by 680C91 (Fig. 6b,c). In primary hippocampal neurons, KYNA reduced dendritic length and branching (Fig. 6d,e) and diminished complexity (Fig. 6f). These abnormalities were rescued by the mTOR agonist MHY1485, indicating that KYNA impairs dendritic development through mTOR suppression. Moreover, MHY1485 treatment of SIRT6-knockdown neurons significantly improved dendritic length, branch numbers, and complexity (Supplementary Fig. 4c–e). In vivo, i.p. administration of MHY1485 for 4 weeks in *Sirt6* cKO mice (Fig. 6g) restored hippocampal levels of p-mTOR and p-p70S6K1, as well as postsynaptic proteins GluA1 and PSD95 (Fig. 6h,i). Golgi staining confirmed that MHY1485 improved dendritic length, branch numbers (Fig. 6j,k and Supplementary Fig. 4f), complexity (Fig. 6l and Supplementary Fig. 4g), spine density (Fig. 6m,n and Supplementary Fig. 4h), and the proportion of mature spines (Fig. 6o and Supplementary Fig. 4i), restoring morphology to WT levels. Behaviorally, MHY1485 enhanced performance in NOR and NLR tests in *Sirt6* cKO mice (Fig. 6p,q). Consistent improvements were observed in the Morris water maze test, with increased platform crossings and time in the target quadrant (Supplementary Fig. 4j). Collectively, these results demonstrate that SIRT6 deficiency leads to TDO2-mediated KYNA accumulation, which suppresses mTOR signaling, thereby impairing synaptic structure and cognitive function.

### Neuronal TDO2 inhibition rescues SIRT6 deficiency-induced impairments by restoring mTOR signaling

**Fig. 7:**
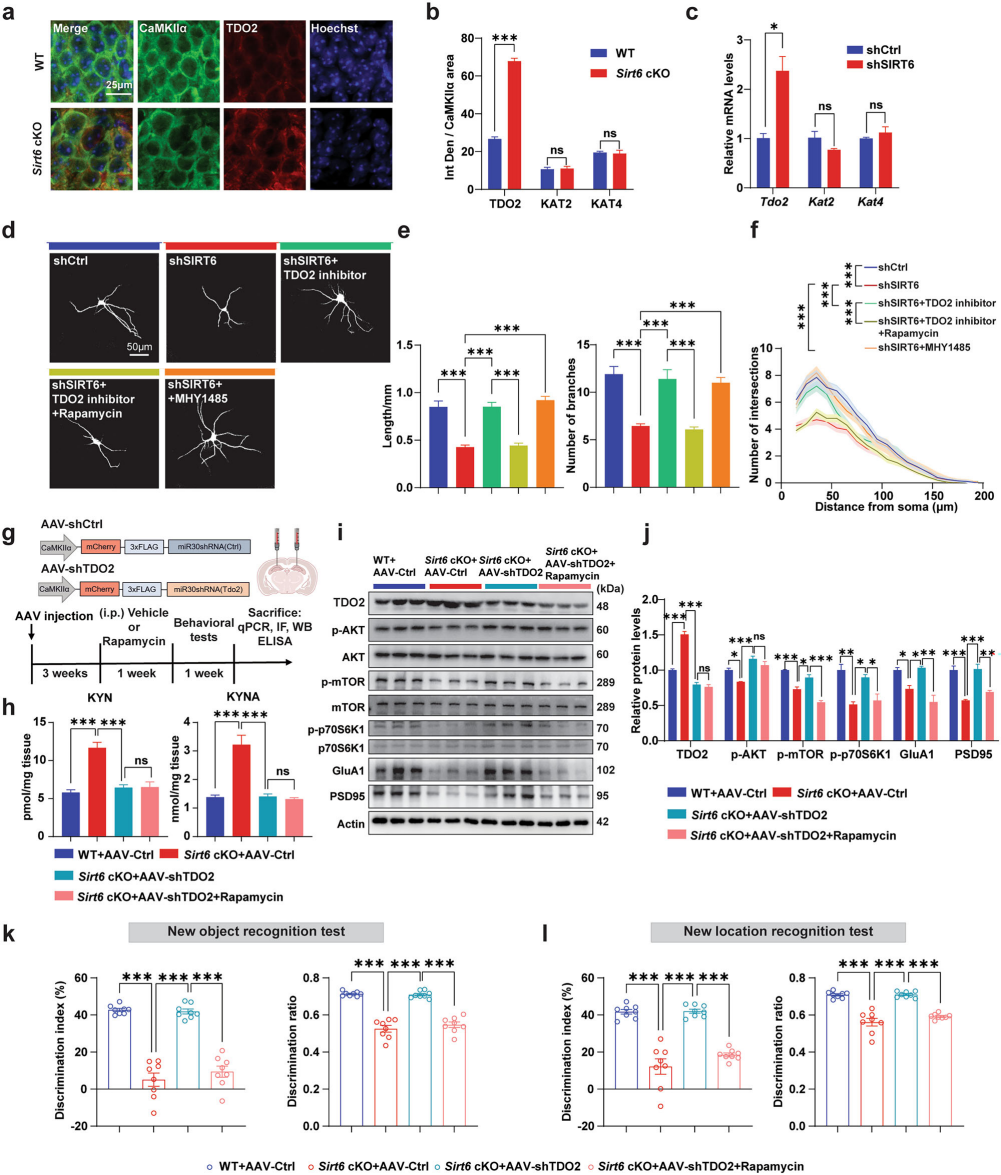
Neuronal Tdo2 inhibition rescues Sirt6 deficiency-induced neuronal and cognitive impairments by restoring mTOR signaling. **a** Representative image of TDO2 in hippocampal CA1 neurons of wild-type (WT) and *Sirt6* conditional knockout (cKO) mice. Scale bar, 25 μm. **b** Quantification of TDO2, KAT2, and KAT4 in hippocampal CA1 neurons of WT and *Sirt6* cKO mice (*n* = 3 mice per group. Statistics are derived from eight slices. **c** Quantitative PCR (qPCR) analysis of *Tdo2*, *Kat2*, and *Kat4* mRNA levels in primary hippocampal neurons following lentiviral-mediated knockdown of SIRT6. **d** Representative images of primary hippocampal neurons immunostained for the neuronal marker MAP2 treated by shCtrl, shSIRT6, shSIRT6 + TDO2 inhibitor (10 μM), shSIRT6 + TDO2 inhibitor (10 μM) + Rapamycin (200 nM), and shSIRT6 + MHY1485 (10 μM). Scale bar, 50 μm. Quantification of total dendritic length and the branch number (part **e**) and Sholl analysis of dendritic morphology (part **f**) of primary hippocampal neurons from the five groups (*n* = 20 neurons from 6–8 mice). **g** Schematic of the experimental timeline. **h** Enzyme-linked immunosorbent assay (ELISA) quantification of hippocampal kynurenine (KYN) and kynurenic acid (KYNA) levels in WT + AAV-Ctrl, *Sirt6* cKO + AAV-Ctrl, *Sirt6* cKO + AAV-shTDO2, and *Sirt6* cKO + AAV-shTDO2 + Rapamycin (5 mg/kg) mice (*n* = 6 mice per group). Representative western blot (WB) images (part **i**) and quantification (part **j**) of TDO2, p-AKT/AKT, p-mTOR/mTOR, p-p70S6K1/p70S6K1, GluA1, and PSD95 levels in the hippocampus from four groups (*n* = 3 mice per group). Discrimination index and discrimination ratio of the novel object recognition test (part **k**) and novel location recognition test (part **l**) across the four groups (*n* = 8 mice/5 litters per group). The data are shown as mean ± SEM. Two-tailed Student’s *t* test in parts **b** and **c**; one-way analysis of variance with Tukey’s multiple comparisons test in parts **e**, **h**, and **j**; two-way analysis of variance followed by Sidak’s multiple comparisons test was used for multiple comparisons in part **f**; linear mixed-effects model in parts **k** and **l**. *P < 0.05, **P < 0.01, and ***P < 0.001. AAV, adeno-associated virus; i.p., intraperitoneal; ns, not significant; PSD, postsynaptic density.

We investigated the SIRT6–TDO2–KYNA–mTOR regulatory axis in neurons. IF staining showed expression of TDO2, KAT2, and KAT4 in hippocampal CaMKIIα^+^ neurons (Fig. 7a and Supplementary Fig. 5a). SIRT6 KO specifically upregulated TDO2 expression, but not KAT2 or KAT4 (Fig. 7b). Consistent with this, SIRT6 knockdown in primary cultured hippocampal neurons elevated *Tdo2* levels without altering *Kat2* or *Kat4* expression (Fig. 7c). Functionally, neuronal SIRT6 knockdown reduced dendritic length, branching, and complexity. TDO2 inhibition completely rescued these morphological deficits, whereas the mTOR inhibitor rapamycin blocked this rescue. Conversely, the mTOR agonist MHY1485 restored normal morphology despite SIRT6 knockdown, confirming a neuronal SIRT6/TDO2/mTOR regulatory axis (Fig. 7d–f). To validate this pathway in vivo, we stereotactically injected a CaMKIIα promoter-driven TDO2 knockdown AAV virus into the hippocampal CA1 region of *Sirt6* cKO mice, combined with rapamycin treatment (Fig. 7g and Supplementary Fig. 5b). qPCR confirmed efficient TDO2 knockdown without compensatory IDO1/IDO2 changes (Supplementary Fig. 5c). Enzyme-linked immunosorbent assay showed that neuronal TDO2 knockdown fully reversed SIRT6 deficiency-induced increases in hippocampal KYN/KYNA, an effect unaffected by rapamycin (Fig. 7h). SIRT6 deficiency also reduced phosphorylation of mTOR pathway proteins and synaptic markers. Neuronal TDO2 knockdown rescued these deficits, whereas rapamycin co-treatment re-suppressed their expression (Fig. 7i,j). Behaviorally, *Sirt6* cKO mice exhibited learning and memory deficits, which were reversed by neuronal Tdo2 knockdown. Crucially, co-administration of rapamycin fully blocked this rescue (Fig. 7k,l). Collectively, these metabolite, protein, and behavioral results support the regulatory axis. Notably, as KYN (but not KYNA) crosses the blood–brain barrier, neuron-specific perturbation of SIRT6 or TDO2 did not alter peripheral plasma KYN levels (Supplementary Fig. 5d) or the expression of metabolic enzymes in the liver (the major synthesis organ) (Supplementary Fig. 5e), indicating that the observed cerebral metabolic alterations are likely under central regulation. Although potential diffusion of neuronal KYN to glia for KYNA conversion cannot be entirely excluded, our in vitro (pharmacological inhibition of Tdo2) and in vivo (neuron-specific TDO2 knockdown) data indicate that the key initiating regulatory node (SIRT6/TDO2) resides in neurons. Thus, neuron-specific TDO2 inhibition rescues SIRT6 deficiency-induced neuronal and cognitive deficits by restoring mTOR signaling.

To elucidate how KYNA accumulation suppresses mTOR signaling, we first measured hippocampal TRP levels in *Sirt6* cKO mice and found a significant increase (Supplementary Fig. 6a), consistent with previous reports in various SIRT6-deficient cell lines^45^. Polysome profiling in HT22 hippocampal neurons revealed that KYNA treatment did not alter the polysomal distribution of key mTOR pathway mRNAs (mTOR, Raptor, and p70S6K1), indicating that translation initiation was not substantially disturbed (Supplementary Fig. 6b). Exogenous KYNA did not affect total AKT, mTOR, or p70S6K1 protein levels in HT22 cells but significantly reduced their phosphorylation (Supplementary Fig. 6c,d). The finding is consistent with suppressed mTOR pathway phosphorylation in *Sirt6* cKO hippocampi, suggesting that KYNA affects the phosphorylation status, rather than the synthesis, of these kinases. Given that KYNA acts as an endogenous NMDA receptor (NMDAR) antagonist^34^ and that NMDAR activation promotes calcium influx and subsequent AKT phosphorylation^55,56^, we tested whether KYNA inhibits AKT/mTOR signaling via NMDAR blockade. In primary hippocampal neurons, exogenous KYNA reduced dendritic length, branch numbers, and complexity (Supplementary Fig. 6e–g), weakened intracellular calcium signaling (Supplementary Fig. 6h,i), and suppressed mTOR pathway phosphorylation (Supplementary Fig. 6j,k) — all of which were fully reversed by co-treatment with the NMDAR agonist D-cycloserine (DCS), confirming that KYNA acts through NMDAR inhibition. Critically, in SIRT6-knockdown neurons, TDO2 inhibitor treatment rescued the dendritic deficits (Supplementary Fig. 6l–n), impaired calcium signaling (Supplementary Fig. 6o,p), and reduced mTOR phosphorylation (Supplementary Fig. 6q,r); this rescue was completely blocked by the NMDAR antagonist MK-801. Likewise, adding DCS effectively restored neuronal morphology, calcium signaling, and pathway activity in SIRT6-knockdown neurons. Together, these results suggest that downregulation of SIRT6 in neurons specifically upregulates TDO2, leading to KYNA accumulation, which in turn antagonizes NMDAR function and thereby suppresses mTOR pathway phosphorylation.

### Neuronal SIRT6 overexpression rescues FGR-induced synaptic and cognitive deficits

**Fig. 8:**
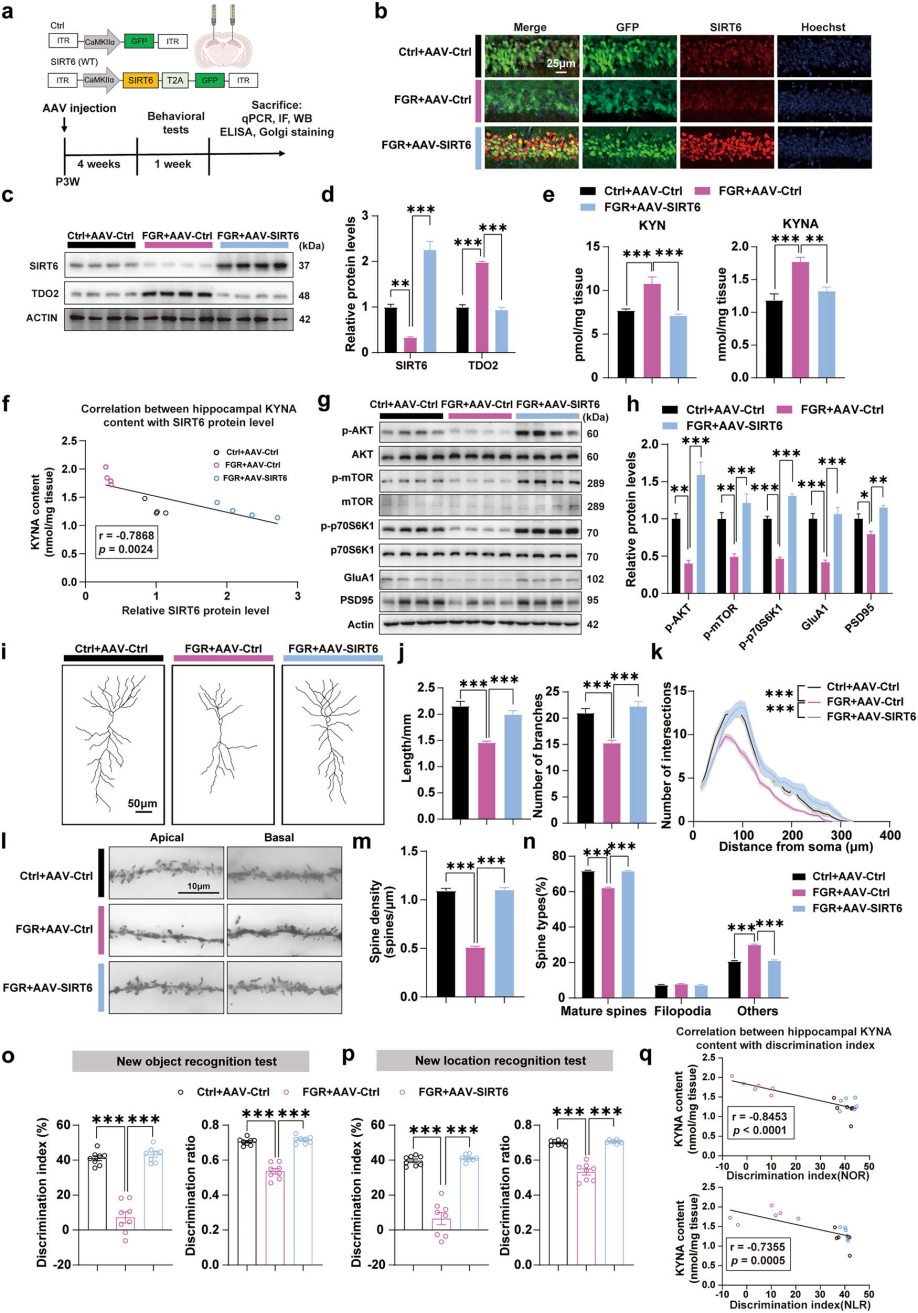
Increasing SIRT6 improves impaired synaptic plasticity and cognition in FGR mice. **a** Schematic of the experimental timeline. **b** Representative immunofluorescence (IF) images showing SIRT6 (red) and GFP (green) in the CA1 region of Ctrl + AAV-Ctrl, FGR + AAV-Ctrl, and FGR + AAV-SIRT6 mice. Scale bar, 25 μm. Representative western blot (WB) images (part **c**) and quantification (part **d**) of SIRT6 and TDO2 protein levels in the hippocampus from three groups (*n* = 4 mice per group). **e** Enzyme-linked immunosorbent assay (ELISA) quantification of hippocampal kynurenine (KYN) and kynurenic acid (KYNA) levels in the three groups (*n* = 6 mice per group). **f** Negative correlation of hippocampal KYNA content with SIRT6 protein level (*n* = 4 mice per group). Representative WB images (part **g**) and quantification (part **h**) of p-AKT/AKT, p-mTOR/mTOR, p-p70S6K1/p70S6K1, GluA1, and PSD95 levels in the hippocampus from three groups (*n* = 4 mice per group). **i** Representative Golgi staining images of hippocampal CA1 neurons from three groups. Scale bar, 50 μm. **j** Quantification of total dendritic length and branch number of the hippocampal CA1 neurons from three groups (*n* = 15 neurons from 3 mice per group). **k** Sholl analysis of dendritic complexity in neurons from three groups (*n* = 15 neurons from 3 mice per group). **l** Representative Golgi staining images of hippocampal CA1 neuronal dendritic spines from three groups. Scale bar, 10 μm. Quantitative analysis of spine density (part **m**) and the percentage of spine types (part **n**) (*n* = 15 neurons from 3 mice per group). Discrimination index and discrimination ratio of the novel object recognition (NOR) test (part **o**) and novel location recognition (NLR) test (part **p**) across the three groups (*n* = 8 mice/5 litters per group). **q** Negative correlation of hippocampal KYNA content with discrimination index in the NOR and NLR tests (*n* = 6 mice per group). The data are shown as mean ± SEM. One-way analysis of variance with Tukey’s multiple comparisons test in parts **d**, **e**, **h**, **j**, **m**, and **n**; two-way analysis of variance followed by Sidak’s multiple comparisons test was used for multiple comparisons in part **k**; linear mixed-effects model in parts **o** and **p**. AAV, adeno-associated virus; FGR, fetal growth restriction; GFP, green fluorescent protein; i.p., intraperitoneal; ns, not significant; PSD, postsynaptic density; P3W, postnatal 3 weeks; qPCR, quantitative PCR. *P < 0.05, **P < 0.01, and ***P < 0.001.

Finally, we investigated whether excitatory neuron-specific overexpression of SIRT6 could reverse cognitive impairments in FGR offspring. Although *Sirt6* and *Tdo2* expression showed no significant differences at E18.5, their levels in the FGR hippocampus diverged by postnatal 3 weeks (P3W), with *Sirt6* decreased and *Tdo2* increased (Supplementary Fig. 7a). This observation guided our selection of P3W for SIRT6-targeted intervention. Using an AAV vector driven by the CaMKIIα promoter (Fig. 8a), we selectively overexpressed SIRT6 in the CA1 region of the hippocampus in FGR mice (Fig. 8b). Four weeks’ post-injection, qPCR and WB analyses confirmed that SIRT6 mRNA and protein levels were significantly reduced in the FGR + AAV-Ctrl group compared with controls, but markedly elevated following AAV-SIRT6 administration (Fig. 8c,d and Supplementary Fig. 7b), confirming successful genetic intervention. Mechanistically, FGR mice exhibited increased hippocampal TDO2 mRNA and protein expression and elevated downstream metabolites KYN and KYNA, all reversed by SIRT6 overexpression (Fig. 8e). Notably, hippocampal KYNA levels were strongly inversely correlated with SIRT6 expression (*r* = −0.7868, *P* = 0.0024; Fig. 8f), reinforcing the gatekeeper role of SIRT6 in TRP catabolism. In addition, SIRT6 restoration rescued mTOR pathway activity, increasing p-AKT, p-mTOR, and p-p70S6K1, alongside synaptic proteins GluA1 and PSD95 (Fig. 8g,h). Golgi analysis revealed that CA1 neurons in FGR mice exhibited substantial dendritic abnormalities, including a 32.42% reduction in total dendritic length, 27.30% fewer branches, reduced dendritic complexity (Fig. 8i–k and Supplementary Fig. 7c,d), lower spine density (Fig. 8l,m and Supplementary Fig. 7e), and a decline in the proportion of mature spines (Fig. 8n and Supplementary Fig. 7f). Remarkably, SIRT6 overexpression significantly reversed all of these morphological deficits. In the NOR and NLR tests, SIRT6 overexpression in FGR mice enhanced both the discrimination index and ratio to control levels (Fig. 8o,p). Moreover, KYNA exhibited strong negative correlations with NOR performance (*r* = −0.8453, *P* < 0.0001) and NLR performance (*r* = −0.7355, *P* = 0.0005), establishing KYNA as a biomarker of SIRT6-modulated cognitive deficits in FGR mice (Fig. 8q). These data demonstrate that SIRT6 overexpression in excitatory neurons is sufficient to counteract FGR-induced synaptic and cognitive deficits by suppressing TDO2/KYNA and rejuvenating mTOR signaling.

## Discussion

FGR induces long-term neurocognitive deficits in offspring, substantially diminishing quality of life and imposing profound socioeconomic burdens. The neuropathological basis of these deficits remains incompletely understood, and no effective therapeutic strategies are currently available. In contrast to previously reported neuroprotective roles of SIRT6, such as maintaining genomic stability, regulating Tau degradation, or modulating APP metabolism to alleviate Alzheimer disease pathology^57–59^, or attenuating postoperative cognitive dysfunction through microglial polarization^60^, our study identifies a distinct mechanism whereby SIRT6 regulates hippocampal synaptic plasticity via TRP metabolism to mitigate cognitive deficits. Mechanistically, SIRT6 deficiency in hippocampal excitatory neurons elevates H3K9ac-dependent TDO2 transcription, diverting TRP metabolism toward KYNA overproduction, which suppresses AKT/mTOR/p70S6K1-dependent synaptic protein synthesis and underlies cognitive deficits in FGR offspring. Our study not only uncovers a novel mechanism through which SIRT6 regulates amino acid metabolism and neural function but also elucidates the molecular bridge linking TRP metabolism dysregulation with synaptic and cognitive deficits in the neuropathology of FGR. This provides new potential therapeutic targets for the targeted intervention of FGR-induced cognitive disorders.

Using a CaMKIIα-Cre-mediated SIRT6 cKO model, we faithfully recapitulated FGR-induced synaptic impairments and cognitive deficits, thereby establishing neuronal SIRT6 as necessary for driving this pathology. Notably, targeted SIRT6 re-expression in FGR offspring fully reversed these deficits, demonstrating its sufficiency in restoring cognitive function. By generating SIRT6 catalytic mutants and combining in vitro primary hippocampal neurons with in vivo AAV-based gene delivery, we demonstrate that SIRT6 modulates synaptic plasticity via its deacetylase activity, but not its ADP-ribosyltransferase activity. These findings are consistent with earlier work showing that deacetylase activity of SIRT6 contributes to dendritic morphogenesis in cultured rat hippocampal neurons^61^. In terms of cellular metabolic regulation, SIRT6 is known to control glucose and lipid homeostasis through its deacetylase function^62–65^. SIRT6 deficiency was also shown to disrupt purine metabolism in cortical astrocytes^23^. A recent study demonstrated that Sirt6 influences sleep in mice and neurodegeneration in *Drosophila* by regulating key enzymes (for example, TDO2 and AANAT) to shift TRP flux toward the KP and away from serotonin/melatonin synthesis^45^. Our study establishes a direct link between excitatory neuron-specific SIRT6 deficiency and cognitive dysfunction mediated by disrupted TRP metabolism in mice. Mechanistically, SIRT6 loss increases H3K9ac, enhancing GR and RNAPII binding to the *Tdo2* promoter and upregulating TDO2. This drives KP flux and KYNA accumulation. Although classical TDO2 regulation involves hormonal/inflammatory signals^66^, and recent studies implicate DNA methylation/H3K27ac^67,68^, our work identifies SIRT6-dependent H3K9ac as a novel epigenetic layer regulating TDO2 expression. Notably, elevated levels of H3K9ac in brain tissue were observed in both broad neural (*Nestin-Cre*) and forebrain excitatory neuron-specific (*Emx1-Cre*) Sirt6 KO models^18,69^. Furthermore, SIRT6, via H3K9ac, targets CHI3L1 in astrocytes and TXNIP in microglia, implicating it in modulating demyelinating and ischemic injury microenvironments^70,71^. Collectively, SIRT6, through mediating H3K9ac, exerts critical regulatory functions across neural cell types in diverse pathophysiological contexts. Our work delineates an SIRT6/H3K9ac–TDO2–KYNA axis in cognitive regulation and expands the role of SIRT6 in neural metabolic networks and disease mechanisms.

The TRP metabolite KYNA displays a dual role in the brain, acting both neuroprotective and neurotoxic depending on the physiological and pathological context^72^. As an endogenous antagonist of NMDA and α7 nicotinic receptors, KYNA can exert neuroprotective effects by alleviating excitotoxicity^73,74^, inhibiting neuroinflammation^72^, and reducing oxidative stress^75^. However, its excessive accumulation can lead to neurotoxicity, particularly impairing cognitive function^30–32,76–78^. Our data reveal that FGR-induced hippocampal KYNA elevation inversely correlates with cognitive performance, and its reduction via SIRT6 overexpression rescues synaptic plasticity and cognitive function. These results highlight the neurotoxic role of KYNA accumulation in FGR-induced cognitive dysfunction. The pathological significance of KYNA extends beyond FGR, with its accumulation documented in aging, Alzheimer disease, schizophrenia, and post-COVID cognitive dysfunction, showing increases in cerebrospinal fluid and brain tissue across these conditions^79–84^. Convergent evidence from independent groups reveals that restricting KYNA accumulation in the brain can effectively alleviate cognitive impairments associated with aging, schizophrenia, and cannabis exposure^85–88^. These findings nominate KYNA as a cross-disease neurotoxic metabolite, offering a novel therapeutic target for broad-spectrum cognitive disorder therapeutics. Notably, our results intersect with emerging paradigms linking early-life environmental insults to elevated brain KYNA levels and subsequent cognitive impairments in adulthood, including prenatal/adolescent tetrahydrocannabinol exposure and lactational lead exposure^85,89,90^. Additionally, exposure to KYNA or its precursor KYN during fetal development, lactation, or adolescence has been shown to trigger cognitive dysfunction in adulthood^91–96^. This conserved mechanism across exposure models identifies KYNA as a metabolic linchpin connecting early-life adverse environments to adult neurodevelopmental disorders.

As a central metabolic sensor, mTOR signaling has a pivotal role in synaptic plasticity and cognition by orchestrating protein synthesis^37^. Although several amino acids and their metabolites such as arginine, leucine, and *S*-adenosylmethionine have been shown to modulate mTOR activity^39^, it remains unknown whether KYNA regulates mTOR signaling or contributes causally to synaptic plasticity. Our study provides the first evidence that KYNA excess leads to mTOR suppression. In both *Sirt6* JO and FGR mouse models, elevated KYNA levels were associated with reduced phosphorylation of the AKT/mTOR/p70S6K1 axis and downregulation of synaptic proteins GluA1 and PSD95. Pharmacological inhibition of TDO2 significantly lowered KYNA levels in the hippocampus of *Sirt6* cKO mice, restoring AKT/mTOR activity, promoting synaptic protein synthesis, and ameliorating cognitive deficits. This mechanism mirrors findings from tumor microenvironments, in which IDO/TDO-mediated mTOR inhibition induces immune tolerance^97,98^. Notably, administration of the mTOR agonist MHY1485 not only reversed the reduction in dendritic length, branching, and complexity induced by KYNA in cultured hippocampal neurons but also ameliorated synaptic loss and cognitive deficits in *Sirt6* cKO mice. KYNA is known to impair NMDAR function through dual pathways: direct antagonism at the glycine site^34,99^ and indirect suppression via α7nAChR inhibition, reducing glutamate release. NMDAR activation promotes AKT phosphorylation via PI3K adapters such as APPL1 and p55PIK^100,101^. Our study demonstrates that SIRT6 inhibition drives dysregulated TRP metabolism and KYNA accumulation, which impairs neuronal morphology and function by antagonizing NMDAR activity, inhibiting calcium influx, and thereby attenuating phosphorylation-dependent activation of the AKT/mTOR pathway. Indeed, multiple studies have associated early-life environmental insults (for example, dexamethasone exposure, synthetic thromboxane, FGR induced by uterine artery ligation, whole-brain irradiation, and lead exposure) with NMDAR dysfunction, resulting in neuronal impairment and cognitive deficits^102–106^. Our study reveals a novel mechanism whereby KYNA suppresses AKT/mTOR signaling, leading to synaptic and cognitive dysfunction.

Together, our findings uncover a new SIRT6–TDO2/KYNA–mTOR regulatory axis that mechanistically links metabolic perturbation to cognitive deficits in FGR offspring, offering new avenues for both mechanistic insight and clinical intervention. Given the availability of pharmacological agents targeting this axis, including SIRT6 activators (MDL-800, NAD⁺ precursors)^107,108^, KYNA-lowering compounds (galantamine, memantine, *N*-acetylcysteine)^33,109^, or mTOR pathway enhancer (NV-5138)^110^, future investigations may pave the way for effective therapeutic strategies to mitigate cognitive impairments associated with FGR and improve long-term quality of life. Beyond FGR, this axis may underlie cognitive dysfunction in aging, neurodegeneration, and psychiatric disorders, where SIRT6, KP metabolites, or mTOR signaling pathway are dysregulated, suggesting that this mechanism may have broader biological significance and implications.

## Supplementary information

Supplementary Information

EMM 20251846RR source data

## Data Availability

RNA-sequencing data have been deposited in the GEO database under the accession number GSE298372. All other data are available within the Source Data file. Source data are provided with this paper.
