# Supplementary material for: Targeting the SIRT6–TDO2/KYNA–mTOR axis rescues synaptic and cognitive deficits in fetal growth restriction offspring
Source: Exp Mol Med. 2026 Jun 11;58(6):1987–2006. doi: 10.1038/s12276-026-01739-7 (PMC13324384; doi:10.1038/s12276-026-01739-7)

Supplementary Information

Targeting the SIRT6-TDO2/KYNA-mTOR axis rescues synaptic and cognitive deficits in fetal growth restriction offspring

Shujuan Chang †, Wen Chen†*, Wei Zhu,

Nana Liu, Yuhang Wang, Jianguo Li, Jiuhong Kang*

Author affiliations:

1. Clinical and Translational Research Center of Shanghai First Maternity and Infant Hospital, Shanghai Key Laboratory of Maternal Fetal Medicine, Shanghai Key Laboratory of Signaling and Disease Research, Frontier Science Center for Stem Cell Research, National Stem Cell Translational Resource Center, School of Life Sciences and Technology, Tongji University, Shanghai, 200092, China. 2. Department of Medicine, Jinggangshan University, Ji'an 343009, China.

†These authors contributed equally: Shujuan Chang, Wen Chen.

Correspondence to: Jiuhong Kang, Wen Chen

E-mail: jhkang@tongji.edu.cn, chenwen1102@tongji.edu.cn

This file includes: Materials and Methods, References, Supplementary Figures. 1-7 Supplementary Tables. 1-2, and Original Data file of Golgi staining.

Materials and Methods

Open field test

Mice were placed in the center of a transparent acrylic chamber (27 × 27 × 20 cm; Med Associates) and allowed to explore freely for 7 minutes. The first 2 minutes were for habituation. Locomotor activity during the last 5 minutes was analyzed using Activity Monitor software.

Elevated plus maze test

The maze consisted of two open arms (35 × 5 × 1 cm) and two closed arms (35 × 5 × 15 cm) elevated 50 cm above the floor. Mice were placed at the center, facing an open arm, and allowed to explore for 5 minutes. Entries (all four paws into an arm) and time spent in each arm were recorded.

Transmission electron microscopy (TEM)

Hippocampal CA1 tissues were dissected into 1 mm^3 cubic blocks and sequentially fixed in 2.5% glutaraldehyde for 24 hr at 4°C, followed by post-fixation in 1% osmium tetroxide for 2 hr at room temperature. After five 10-minute washes with PB buffer, samples underwent graded acetone dehydration with 30%, 50%, 70%, and 90% acetone for 40 minutes each time. Dehydrate 100% acetone 3 times for 10 minutes each. Dehydrated tissues were infiltrated with EPON using acetone-resin series (3:7 ratio) for 4 h and acetone-resin series (7:3 ratio) overnight before final embedding and polymerization at 60°C for 48 hr. Ultrathin sections (50 nm) were cut using a Leica UC7 ultramicrotome, collected on 200-mesh copper grids, and stained with uranyl acetate and lead citrate. Ultrathin sections were examined using a JEM-1230 transmission electron microscope (JEOL), and images were analyzed using RADIUS software (EMSIS). Measurements of PSD-related thickness were performed according

to previously reported methods[1]. Within the RADIUS software, the cytoplasmic outline of the target postsynaptic density (PSD), including associated dense material, was manually traced. The area was measured and recorded. Subsequently, the corresponding postsynaptic membrane contour was traced to form a complete closed region, and the length of this postsynaptic membrane segment was measured and recorded. PSD thickness was calculated using the formula: *Average PSD Thickness = Enclosed Area / Corresponding Postsynaptic Membrane Length*. Quantification of vesicle number was performed according to previously reported methods[2]: Synaptic vesicles within a 0.04 mm^2 area surrounding the synaptic active zone and in the adjacent region of the synaptic terminal were manually counted for each synapse. The final quantified results are presented as the number of vesicles per individual synapse.

Primary hippocampal neuron culture

Hippocampi were dissected from mice on PD1 and minced. Tissues were digested in 0.125% trypsin at 37°C for 20 minutes, with gentle agitation every 5 minutes. The digestion was quenched with 2× volume of DMEM/F12 medium (Gibco, C11330500BT) containing 10% FBS (Gibco, 10270106), and 1% penicillin-streptomycin (Invitrogen, 15140122), followed by centrifugation (1000 rpm, 10 min, 4°C). Cells were resuspended, filtered through a 70 µm strainer, and plated at 4×10^4 cells/well on poly-L-ornithine (Sigma, P4957) and laminin (Sigma, L2020)-coated 24-well plates. After 4 hours, the medium was replaced with serum-free Neurobasal medium (Gibco, 21103049) containing 2% B27 (Invitrogen, 12587010), 1% GlutaMAX (Invitrogen, 35050061), and 1% penicillin-streptomycin. Half of the medium was replaced every 3 days.

Plasmid construction

For gene knockdown, shRNA sequences (targeting or non-targeting control) were

cloned into the pLKO.1 vector (Addgene, 8453) at the AgeI and EcoRI sites (sequences listed in Supplementary Tab. 1). For gene overexpression, the full-length cDNA of mouse *Sirt6* (NM_181586.4) was amplified using KOD DNA polymerase (Genview, GK1101) and cloned into the FUGW lentiviral vector (Addgene, 14883) with C-terminal FLAG tag. SIRT6 mutants, including H133Y, S56Y, R65A, and G60A, were generated via site-directed mutagenesis using QuikChange Kit (Stratagene, 200516). A plasmid encoding luciferase was used as a control vector.

Lentivirus packaging and infection

HEK293FT cells were seeded in 6-well plates at a density of 1.3×10^6 cells/well and cultured in DMEM (Gibco, C11965500BT) supplemented with 10% FBS. On the following day, cells were co-transfected with 1.2 μg target plasmid, 0.9 μg PAX2, and 0.6 μg VSVG using Hieff Trans Liposomal Transfection Reagent (YEASEN, 40802ES03). After 8 hours, the medium was replaced, and viral supernatants were harvested at 48 hours post-transfection, filtered (0.45 μm), and concentrated using 5× Lentivirus Concentrator (ExCell Bio, EMB810A-1). Virus pellets were resuspended in 100 μL PBS and applied to cultured hippocampal neurons at 5 μL/well in 24-well plates. Medium was replaced after 48 hours.

Enzyme-linked immunosorbent assay (ELISA)

Hippocampal CA1 tissues (one unilateral hippocampus per sample) were rapidly dissected on ice-chilled platforms, weighed, and homogenized in 100 μL ice-cold PBS (pH 7.4) using a pre-cooled 1 mL glass homogenizer. Homogenates were centrifuged at 2000 rpm for 20 minutes at 4°C, and supernatants were collected for subsequent analysis. Whole blood was collected from mice using anticoagulant tubes, allowed to stand at room temperature for 20 minutes, and then centrifuged at 4°C and 3000 rpm for 20 minutes. The upper plasma layer was collected for subsequent analysis.

Quantification of KYN and KYNA was performed using ELISA kits (KYN: RJ17525; KYNA: RJ17528; Shanghai Ren Jie Biotechnology) following manufacturer protocols with critical modifications. For the assay, TRP and KYN standards were prepared at concentrations of 0, 30, 60, 120, 240, and 480 pg/mL, while KYNA standards were prepared at 0, 25, 50, 100, 200, and 400 μmol/L. Pre-coated microplates were pre-equilibrated to room temperature for 20 min after 4°C storage. Samples underwent 1:5 volumetric dilution (10 μL supernatant + 40 μL dilution buffer) prior to loading. All samples and standards were assayed in duplicate. In the antigen binding, 50 μL of standards or diluted samples was added to each well, followed by the addition of 100 μL HRP-conjugated detection antibody. After sealing the plate and incubating at 37°C for 60 minutes, the liquid was discarded and the plate was blotted dry, followed by five washes with 350 μL wash buffer (1-minute incubation per wash). Subsequently, 50 μL each of Chromogen Solution A and B were added to each well for 15-minute at 37°C protected from light, after which 50 μL stop solution was added and the optical density (OD) was measured at 450 nm using SpectraMax iD3 Multi-Mode Microplate Reader (Molecular Devices). A standard curve was generated by plotting the standard concentrations (X-axis) against their corresponding OD values (Y-axis), and sample concentrations were calculated based on the regression equation. For tissue, the concentration was normalized to tissue weight and presented as picomole per mg tissue or nanomoles per mg tissue[3]. For plasma, the concentration was normalized to fluid volume and expressed as picomoles per milliliter[4].

HT22 cell culture

HT22 cells（Shanghai Zhong Qiao Xin Zhou Biotechnology, ZQ0476）were cultured in DMEM supplemented with 10% FBS and 1% penicillin-streptomycin at 37℃ and

5% CO_2.

Polysome profile

This method was performed as previously reported[5], with minor modifications. HT22 cells were seeded in 10 cm dishes at a density of 3 × 10^6 cells/dish and cultured in DMEM supplemented with 10% fetal bovine serum (FBS). Cells were treated with either DMSO or 1 mM kynurenic acid (KYNA) for 48 hours. Subsequently, cells were treated with 100 μg/mL cycloheximide (CHX) (Selleck, S7418) for 10 minutes in a 37°C incubator. Cells were then trypsinized, collected, and washed twice with ice-cold phosphate-buffered saline (PBS) containing 100 μg/mL CHX, followed by centrifugation at 1,000 rpm for 5 min at 4°C. The supernatant was discarded, and the cell pellet was resuspended in 500 μL of polysome extraction buffer (20 mM Tris-HCl pH 7.5, 100 mM KCl, 5 mM $MgCl_2$, and 0.5% NP-40) supplemented with 100 μg/mL CHX, 1X protease inhibitor cocktail, and 1X RNase inhibitor. The cells were thoroughly pipetted to disrupt the pellet and incubated on ice for 10 min. The lysate was then centrifuged at 13,000 rpm for 10 min at 4°C. The resulting supernatant was carefully layered onto the top of a pre-formed 10%–50% (w/v) sucrose density gradient in a 13.2 mL centrifuge tube (Beckman Coulter, 331372). Gradients were ultracentrifuged at 39,000 rpm for 2 hours at 4°C using an SW41Ti swinging-bucket rotor (Beckman Coulter, 333136). Following centrifugation, gradients were fractionated using an automated density gradient fractionation system (BIOCOMP, Piston Gradient Fractionator), collecting fractions into twelve 1.5 mL centrifuge tubes. RNA was extracted from each fraction. The polysome profile was analyzed by qPCR. Primer sequences[6] used for qPCR are listed in Supplementary Table 1.

Intraperitoneal injection of Rapamycin

Rapamycin (Selleck, S1039) was dissolved in DMSO to a 10.94 mM stock solution,

then diluted in a vehicle composed of 10% DMSO, 40% PEG300, 5% Tween-80, and 45% saline to achieve a working concentration of 1 μg/μL. Mice received intraperitoneal (i.p.) injections at a dosage of 5 mg/kg/day for 1 week before behavioral tests[7].

In vitro pharmacological treatments and compound preparation

The following compounds were used for *in vitro* treatments, with details on their source, dissolution, stock preparation, and final working concentration in serum-free Neurobasal medium. KYNA (kynurenic acid; Selleck, S4719): Dissolved in 1 N NaOH, diluted to 0.5 M in PBS, and and used at a final concentration of 1 mM[8]. DCS (D-cycloserine; Selleck, S1998): Dissolved in DMSO, diluted to 20 mM, and applied at a final concentration of 20 μM[9]. TDO2 inhibitor 680C91 (Selleck, S8997): Dissolved in DMSO, diluted to 10 mM, and used at a final concentration of 10 μM[10]. MK801 (Dizocilpine; Selleck, S2876): Dissolved in DMSO, diluted to 10 mM, and applied at a final concentration of 10 μM[11]. Rapamycin (Selleck, S1039): Dissolved in DMSO, diluted to 2 mM, and used at a final concentration of 200 nM[12]. MHY1485 (Selleck, S7811): Dissolved in DMSO, diluted to 10 mM, and applied at a final concentration of 10 μM[13].

Calcium imaging

Experimental procedures and analytical methods were performed as previously reported[14]. Primary neuronal cells were washed once with artificial cerebrospinal fluid (ACSF) (126 mM NaCl, 2.5 mM KCl, 26 mM $NaHCO_3$, 2 mM $CaCl_2$, 2 mM $MgCl_2$, 1.25 mM NaH_2PO_4, and 10 mM glucose), followed by incubation in ACSF containing 5 μM Fluo-4/AM (Invitrogen, F14217) at 37°C for 1 hour. Time-lapse calcium imaging was conducted using a Leica TCS SP8 confocal microscope with a 40 × objective lens in ACSF, with images acquired every 2 seconds for a total of 200 frames. Regions of

interest (ROIs) were manually selected using Image J software, and the average gray value was taken as the fluorescence intensity (F). The minimum fluorescence value of the target neuronal cells throughout the imaging process was defined as F basal, while the average fluorescence value of the background region across all frames was defined as F background. The calculation method for fluorescence change was: $\Delta F/F = (F - F_{basal}) / F_{background}$. $\Delta F > 0.2$ was defined as a calcium spike, and neurons with one or more calcium spikes were regarded as signal-responsive cells.

References

1 Dosemeci, A. *et al.* Glutamate-induced transient modification of the postsynaptic density. *Proc Natl Acad Sci U S A* **98**, 10428-10432, doi:10.1073/pnas.181336998 (2001).

2 Wang, Y. *et al.* KDM6B cooperates with Tau and regulates synaptic plasticity and cognition via inducing VGLUT1/2. *Mol Psychiatry* **27**, 5213-5226, doi:10.1038/s41380-022-01750-0 (2022).

3 Lu, L. *et al.* Mechanism of Treadmill Exercise Combined with Rich Environmental Stimulation to Improve Depression in Post-stroke Depression Model Rats. *Actas Esp Psiquiatr* **52**, 693-704, doi:10.62641/aep.v52i5.1771 (2024).

4 Hasırcı Bayır, B. R. *et al.* The role of kynurenine pathway metabolites in clinical features and chronic migraine. *Rev Neurol (Paris)*, doi:10.1016/j.neurol.2025.11.006 (2025).

5 Lyu, Y. *et al.* Cpmer: A new conserved eEF1A2-binding partner that regulates Eomes translation and cardiomyocyte differentiation. *Stem Cell Reports* **17**,

1154-1169, doi:10.1016/j.stemcr.2022.03.006 (2022).

6 Ravi, V. *et al.* SIRT6 transcriptionally regulates global protein synthesis through transcription factor Sp1 independent of its deacetylase activity. *Nucleic Acids Res* **47**, 9115-9131, doi:10.1093/nar/gkz648 (2019).

7 Ehninger, D. *et al.* Reversal of learning deficits in a Tsc2+/- mouse model of tuberous sclerosis. *Nat Med* **14**, 843-848, doi:10.1038/nm1788 (2008).

8 Langner, E. *et al.* Kynurenic Acid Induces Impairment of Oligodendrocyte Viability: On the Role of Glutamatergic Mechanisms. *Neurochem Res* **42**, 838-845, doi:10.1007/s11064-016-2009-7 (2017).

9 Vestring, S. *et al.* D-Cycloserine enhances the bidirectional range of NMDAR-dependent hippocampal synaptic plasticity. *Transl Psychiatry* **14**, 18, doi:10.1038/s41398-023-02725-7 (2024).

10 Cuartero, M. I. *et al.* L-kynurenine/aryl hydrocarbon receptor pathway mediates brain damage after experimental stroke. *Circulation* **130**, 2040-2051, doi:10.1161/circulationaha.114.011394 (2014).

11 Nasr, P. *et al.* Influence of cytosolic and mitochondrial Ca2+, ATP, mitochondrial membrane potential, and calpain activity on the mechanism of neuron death induced by 3-nitropropionic acid. *Neurochem Int* **43**, 89-99, doi:10.1016/s0197-0186(02)00229-2 (2003).

12 Beccari, S. *et al.* Microglial phagocytosis dysfunction in stroke is driven by energy depletion and induction of autophagy. *Autophagy* **19**, 1952-1981, doi:10.1080/15548627.2023.2165313 (2023).

13 Huang, X. *et al.* Genetic Analysis of Neurite Outgrowth Inhibitor-Associated Genes in Parkinson's Disease: A Cross-Sectional Cohort Study. *CNS Neurosci Ther* **30**, e70070, doi:10.1111/cns.70070 (2024).

14 Yuan, F. *et al.* Modeling disrupted synapse formation in wolfram syndrome using hESCs-derived neural cells and cerebral organoids identifies Riluzole as a therapeutic molecule. *Mol Psychiatry* **28**, 1557-1570, doi:10.1038/s41380-023-01987-3 (2023).

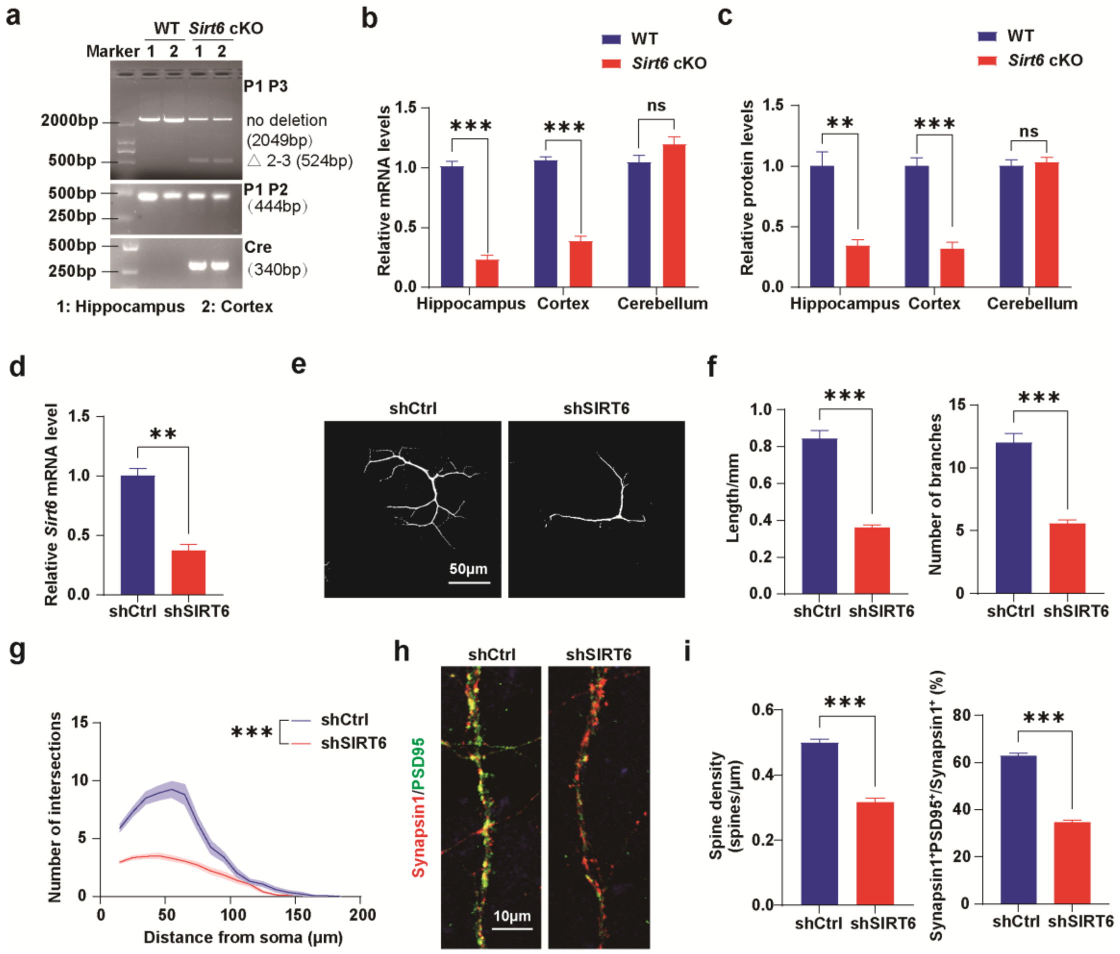

Supplementary Fig. 1 Identification of *Sirt6* cKO mice and the impact of SIRT6 knockdown on dendritic and synaptic morphology in hippocampal neurons.

(a) Representative genotype identification map of transgenic *Sirt6* cKO mice. (b) qPCR analysis of *Sirt6* mRNA level in the hippocampus, cortex and cerebellum from WT and *Sirt6* cKO mice (n = 6 mice per group). (c) Quantification of SIRT6 protein level in the hippocampus, cortex and cerebellum from WT and *Sirt6* cKO mice (n = 4 mice per

group). (d) qPCR analysis of *Sirt6* mRNA level in cultured hippocampal neurons lentiviral infection with shCtrl or shSIRT6 constructs. (e) Representative images of cultured hippocampal neurons immunostained for the neuronal marker MAP2 from shCtrl and shSIRT6 groups. Scale bar, 50 μm. (f) Quantification of dendritic length and branch numbers of primary hippocampal neurons from two groups (n = 20 neurons from 6-8 mice). (g) Sholl analysis of dendritic morphology of primary hippocampal neurons from two groups (n = 20 neurons from 6-8 mice). (h) Representative images showing synaptic sites in primary hippocampal neurons from two groups. Scale bar, 10 μm. (i) Quantification of total spine density ($Synapsin1^{+}$/μm) and intact synaptic contacts ($Synapsin1^{+}PSD95^{+}$/ $Synapsin1^{+}$) in hippocampal neurons from two groups (n = 20 neurons from 6-8 mice). The data are shown as mean ± SEM. Two-tailed Student's t test in (b), (c), (d), (f), (i); two-way ANOVA followed by Sidak's multiple comparisons test was used for multiple comparisons in (g). $^{**}p < 0.01$, $^{***}p < 0.001$, ns, not significant.

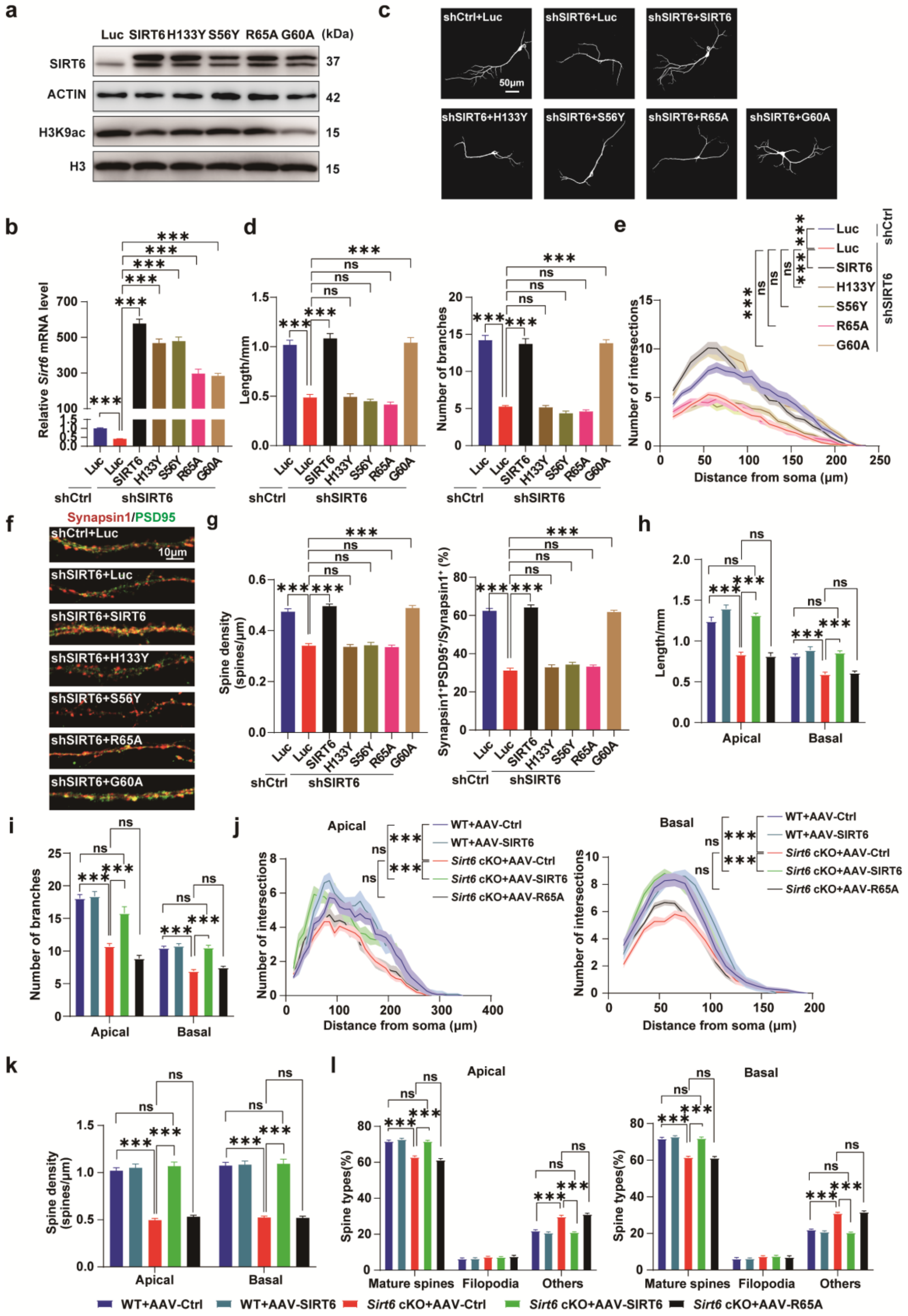

Supplementary Fig. 2 Effects of SIRT6 mutations on dendritic and synaptic properties in hippocampal neurons.

(a) WB analysis of SIRT6 WT and mutant protein expression in 293T cells. 293T cells were seeded at a density of 3×10^5 cells/well in 6-well plates. The transfection was performed with 1.2 μg of target plasmid at the next day and cells were harvested 48 hours post-treatment. (b) qPCR analysis of *Sirt6* mRNA level in the cultured hippocampal neurons of shCtrl+Luc, shSIRT6+Luc, shSIRT6+SIRT6, shSIRT6+H133Y, shSIRT6+S56Y, shSIRT6+R65A, shSirt6+G60A groups. (c) Representative images of the cultured hippocampal neurons immunostained for the neuronal marker MAP2 from the seven groups. Scale bar, 50 μm. (d) Quantification of dendritic length and branch numbers in primary hippocampal neurons from seven groups (n = 20 neurons from 6-8 mice). (e) Sholl analysis of dendritic morphology in primary hippocampal neurons from seven groups (n = 20 neurons from 6-8 mice). (f) Representative images showing synaptic sites in primary hippocampal neurons from seven groups. Scale bar, 10 μm. (g) Quantification of total spine density ($Synapsin1^+$/ μm) and intact synaptic contacts ($Synapsin1^+PSD95^+$/ $Synapsin1^+$) in hippocampal neurons from seven groups (n = 20 neurons from 6-8 mice). (h, i) Quantification of the dendritic length (h) and branch numbers (i) in the apical and basal dendrites of hippocampal CA1 neurons from WT+AAV-Ctrl, WT+AAV-SIRT6, *Sirt6* cKO+AAV-Ctrl, *Sirt6* cKO+AAV-SIRT6 and *Sirt6* cKO+AAV-R65A groups (n = 15 neurons from 3 mice per group). (j) Sholl analysis of dendritic complexity in in the apical and basal dendrites of hippocampal CA1 neurons from five groups (n = 15 neurons from 3 mice per group). (k, l) Quantitative analysis of spine density (k) and spine type distribution (l) in the apical and basal dendrites of CA1 neurons from five groups (n = 15 neurons from 3 mice per group). The data are shown as mean ± SEM. One-way ANOVA with Tukey's multiple comparisons test in (b), (d), (g), (h), (i), (k), (l); two-way ANOVA followed by Sidak's multiple comparisons test was used for multiple comparisons in

(e), (j). ***$p < 0.001$, ns, not significant.

a

Trancription Factor	Score	Relative score	Sequence ID	Predicted
MA0113.2.NR3C1	10.33503	0.877206	mm10_Tdo2_promoter	agaacaagctgttgc
MA0113.2.NR3C1	9.693186	0.86921567	mm10_Tdo2_promoter	gtagcacactgtacc
MA0113.1.NR3C1	10.85479	0.8458887	mm10_Tdo2_promoter	agcaacagcttgttctag
MA0113.2.NR3C1	6.868081	0.83404577	mm10_Tdo2_promoter	gggtctacaggttct
MA0113.2.NR3C1	6.849885	0.83381927	mm10_Tdo2_promoter	agaacacaaagccat
MA0113.2.NR3C1	6.414661	0.8284012	mm10_Tdo2_promoter	gggagagagagatct
MA0113.2.NR3C1	6.163323	0.8252722	mm10_Tdo2_promoter	gcaacagcttgttct
MA0113.2.NR3C1	5.319711	0.81477004	mm10_Tdo2_promoter	gccacacccagtcca
MA0113.2.NR3C1	4.875337	0.809238	mm10_Tdo2_promoter	agaccaggctggcct
MA0113.2.NR3C1	4.731268	0.8074445	mm10_Tdo2_promoter	gacacagacagtgtc
MA0113.2.NR3C1	4.563437	0.8053552	mm10_Tdo2_promoter	aagacagaagttcca
MA0113.2.NR3C1	4.450219	0.80394566	mm10_Tdo2_promoter	agtgcaaattgtcta

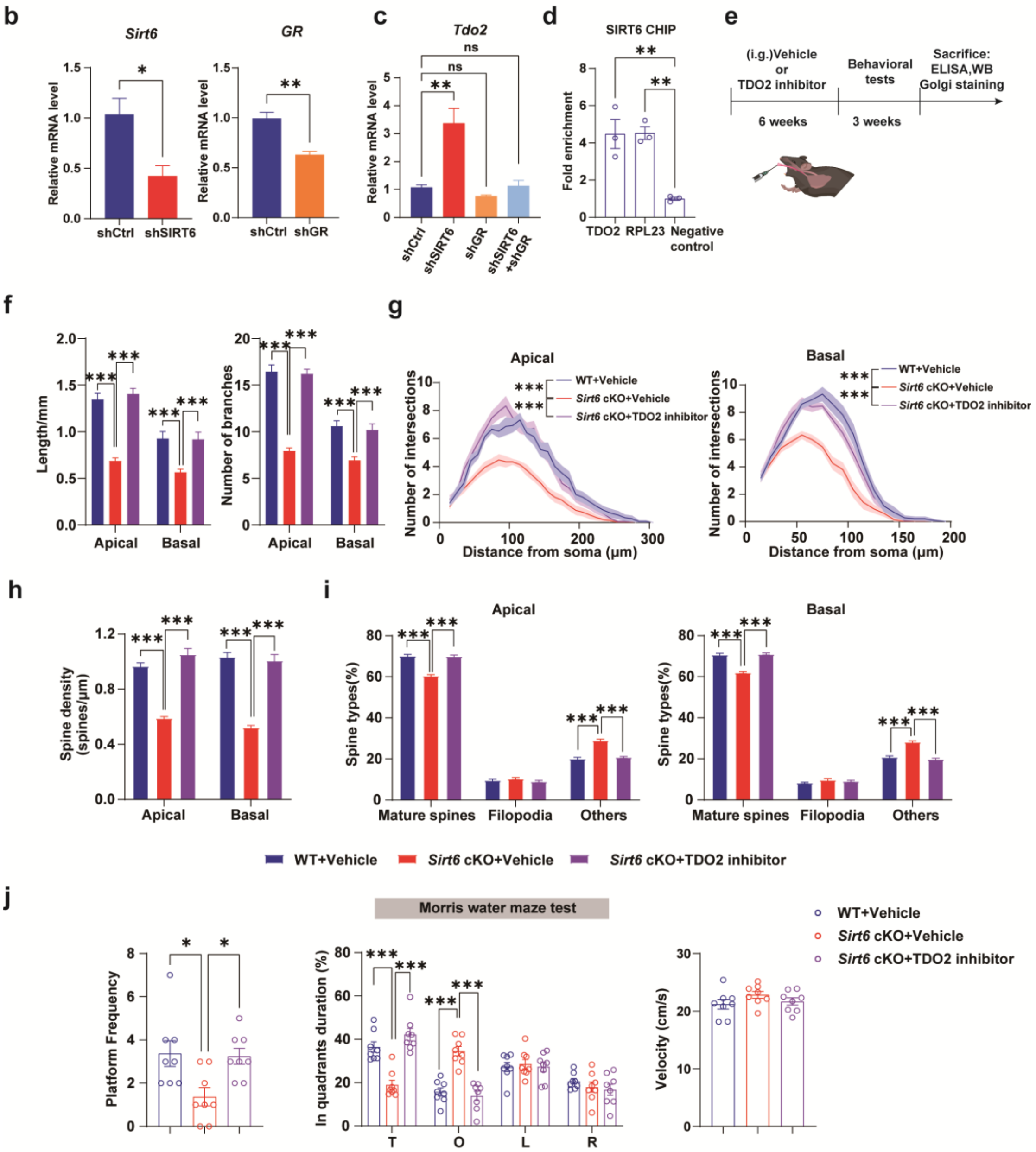

Supplementary Fig. 3 Role of GR in SIRT6-mediated TDO2 regulation, and rescue of morphology and behavior in *Sirt6* cKO mice by TDO2 inhibitor.

(a) JASPAR database (http://jaspar.genereg.net/) prediction results of GR binding sites in the TDO2 promoter region. (b) qPCR analysis confirms specific knockdown of *Sirt6* mRNA (with shSIRT6) and *GR* mRNA (with shGR) in lentivirus-infected HT22 cells versus shCtrl. (c) qPCR analysis of Tdo2 mRNA in HT22 cells with SIRT6 and/or GR knockdown via lentiviral infection. (d) ChIP-qPCR preformed in WT mice for SIRT6. RPL23 as a positive control of binding and intergenic region is used as negative control. The SIRT6 ChIP results were normalized to IgG ChIP and represent fold enrichment from the IgG ChIP. (e) Schematic of the experimental timeline for TDO2 inhibitor (i.g.) administration (7.5 mg/kg). (f) Quantification of the dendritic length and branch number in the apical and basal dendrites of hippocampal CA1 neurons from WT+Vehicle, *Sirt6* cKO+Vehicle, and *Sirt6* cKO+TDO2 inhibitor groups (n = 15 neurons from 3 mice per group). (g) Sholl analysis of dendritic complexity in in the apical and basal dendrites of hippocampal CA1 neurons from three groups (n = 15 neurons from 3 mice per group). (h, i) Quantitative analysis of spine density (h) and the percentage of spine types (i) in the apical and basal dendrites of CA1 neurons from three groups (n = 15 neurons from 3 mice per group). (j) Platform frequency, the percentage of duration in four quadrants and swim velocity during the Morris water maze test from three groups (n = 8 mice/5 litters per group). The data are shown as mean ± SEM. Two-tailed Student's t test in (b); one-way ANOVA with Tukey's multiple comparisons test in (c), (d), (f), (h), (i); two-way ANOVA followed by Sidak's multiple comparisons test was used for multiple comparisons in (g); linear mixed-effects model in (j). *$p < 0.05$, ***$p < 0.001$.

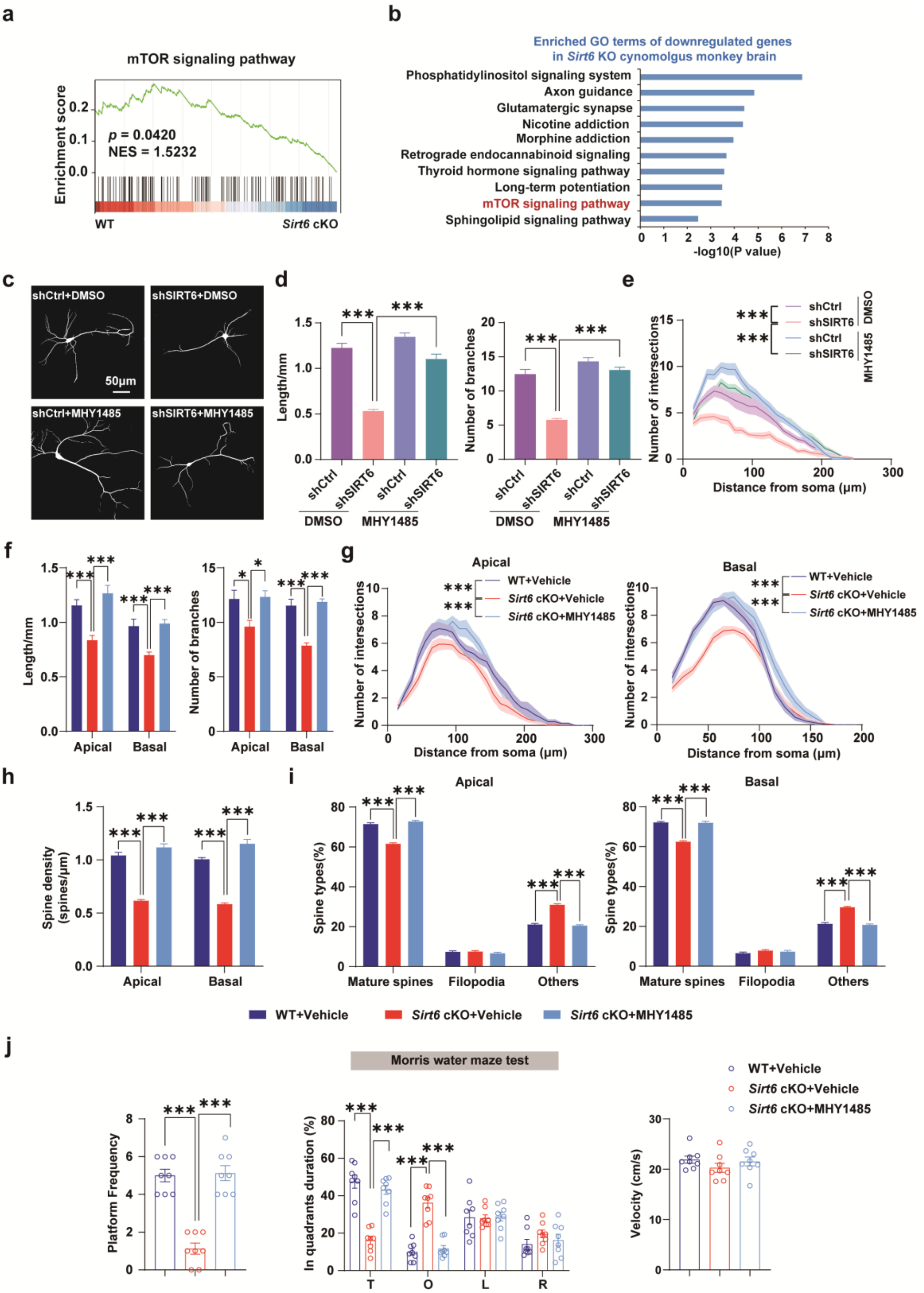

Supplementary Fig. 4 MHY1485 treatment rescues dendritic morphology and synaptic function in hippocampal neurons with SIRT6 knockdown.

(a) GSEA showing negative enrichment of mTOR signaling pathway in the

hippocampus of *Sirt6* cKO mice. (b) GO analysis of downregulated genes in the brains of *Sirt6* KO cynomolgus monkeys. (c) Representative images of the cultured hippocampal neurons immunostained for the neuronal marker MAP2 from shCtrl+DMSO, shSIRT6+DMSO, shCtrl+MHY1485, shSIRT6+MHY1485 groups. Scale bar, 50 μm. (d) Quantification of dendritic length and branch numbers in primary hippocampal neurons from the four groups (n = 20 neurons from 6-8 mice). (e) Sholl analysis of dendritic complexity in primary hippocampal neurons across four groups (n = 20 neurons from 6-8 mice). (f) Quantification of the dendritic length and branch numbers in the apical and basal dendrites of hippocampal CA1 neurons from WT+Vehicle, *Sirt6* cKO+Vehicle, and *Sirt6* cKO+MHY1485 groups (n = 15 neurons from 3 mice per group). (g) Sholl analysis of dendritic complexity in in the apical and basal dendrites of hippocampal CA1 neurons from three groups (n = 15 neurons from 3 mice per group). (h, i) Quantitative analysis of spine density (h) and the percentage of spine types (i) in the apical and basal dendrites of CA1 pyramidal neurons from three groups (n = 15 neurons from 3 mice per group). (j) Platform frequency, the percentage of duration in four quadrants and swim velocity during the Morris water maze test from three groups (n = 8 mice/5 litters per group). The data are shown as mean ± SEM. One-way ANOVA with Tukey's multiple comparisons test in (d), (f), (h), (i); two-way ANOVA followed by Sidak's multiple comparisons test was used for multiple comparisons in (e), (g); linear mixed-effects model in (j). $^{*}p < 0.05$, $^{***}p < 0.001$.

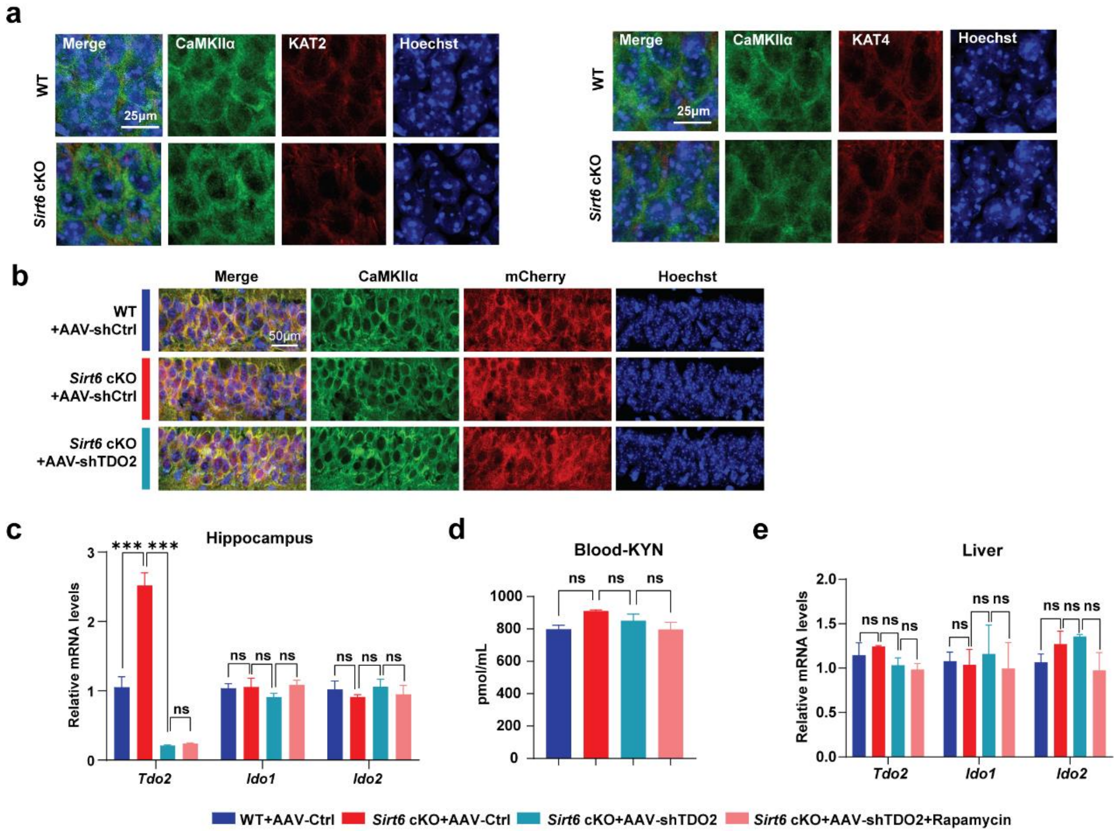

Supplementary Fig. 5 Analysis of hippocampal KATs expression, peripheral blood KYN and liver metabolic enzyme levels in SIRT6 KO mice.

(a) Representative images of KAT2 and KAT4 in hippocampal CA1 neurons of WT and *Sirt6* cKO mice. Scale bar, 25 μm. (b) Representative immunofluorescence images showing mCherry (red) and CaMKIIα (green) in the CA1 region of WT+AAV-Ctrl, *Sirt6* cKO+AAV-Ctrl and *Sirt6* cKO+AAV-shTDO2. Scale bar, 50 μm. (c) qPCR analysis of hippocampal *Tdo2, Ido1* and *Ido2* mRNA levels from the four groups (n = 3 mice per group). (d) ELISA quantification of KYN levels in blood from the four groups (n = 6 mice per group). (e) Quantitative PCR analysis of *Tdo2, Ido1* and *Ido2* mRNA levels in liver tissues from the four groups (n = 3 mice per group). The data are shown as mean ± SEM. One-way ANOVA with Tukey's multiple comparisons test in (c), (d), (e). ***$p < 0.001$, ns, not significant.

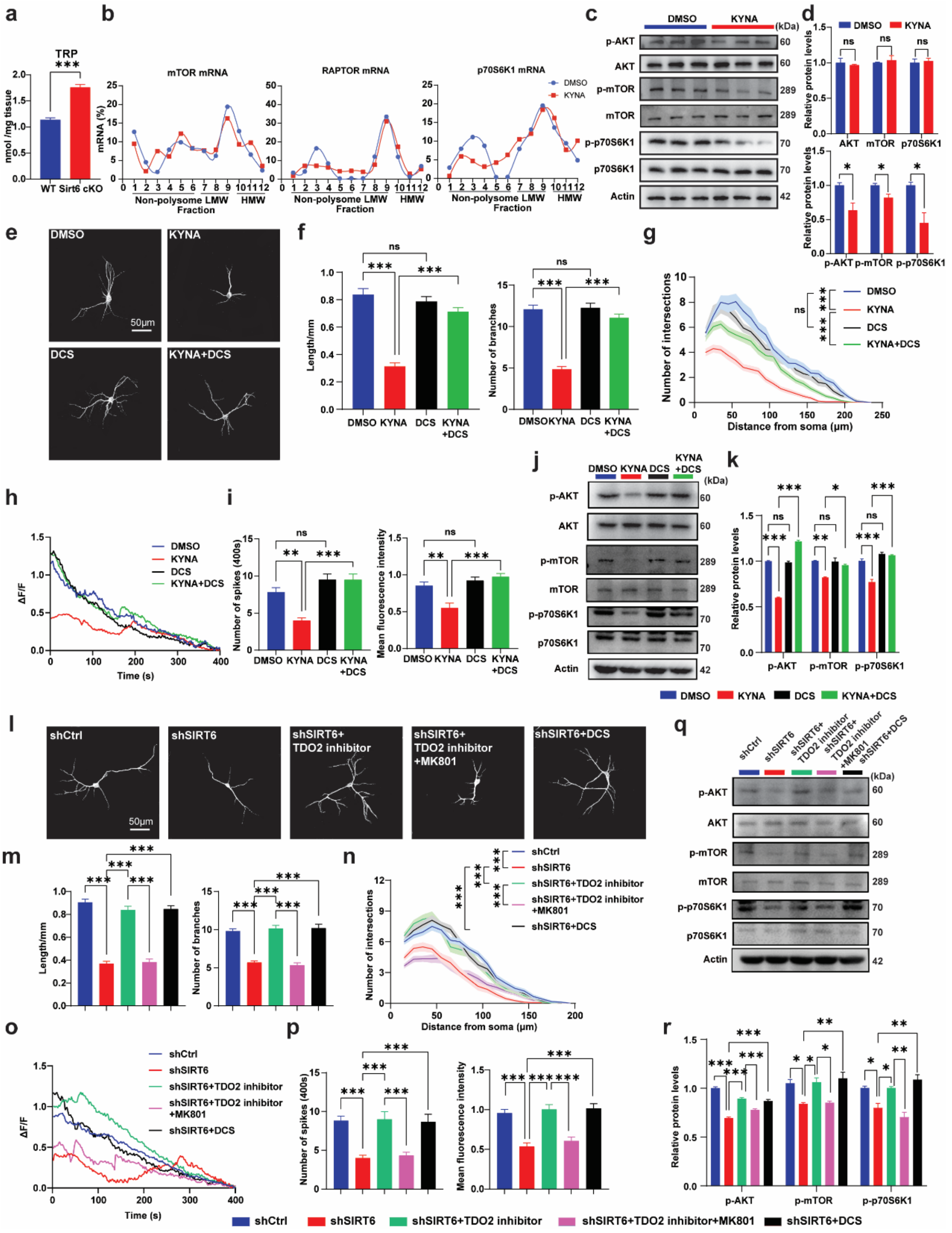

Supplementary Fig. 6 KYNA accumulation suppresses mTOR phosphorylation by inhibiting NMDAR function.

(a) ELISA quantification of hippocampal TRP levels in WT and *Sirt6* cKO mice (n = 6 per group). (b) Percentage of mTOR, RAPTOR and p70S6K1 mRNAs in the gradient total RNA, as measured by qRT-PCR in each fraction collected from the polysome

profiling assay. LMW, low molecular weight; HMW, high molecular weight. (c, d) Representative WB images (c) and quantification (d) p-AKT/AKT, p-mTOR/mTOR and p-p70S6K1/p70S6K1 protein levels in HT22 cells treated by DMSO (Ctrl) and KYNA (1mM). (e) Representative images of cultured hippocampal neurons immunostained for the neuronal marker MAP2 treated by DMSO (Ctrl), KYNA (1mM), DCS (20 μM) and KYNA (1mM) +DCS (20 μM). Scale bar, 50 μm. (f, g) Quantification of total dendritic length and the branch number (f) and sholl analysis of dendritic morphology (g) of primary hippocampal neurons from the four groups (n = 20 neurons from 6-8 mice). (h) Representative single cell traces of intracellular spontaneous calcium activity of the four groups. (i) Calcium spike frequency and mean fluorescence intensity from the four groups (n = 6 fields from 3 independent experiments). (j, k) Representative WB images (j) and quantification (k) p-AKT/AKT, p-mTOR/mTOR and p-p70S6K1/p70S6K1 protein levels from the four groups. (l) Representative images of cultured hippocampal neurons immunostained for the neuronal marker MAP2 treated by shCtrl, shSIRT6, shSIRT6+TDO2 inhibitor (10 μM), shSIRT6+TDO2 inhibitor (10 μM) +MK801 (10 μM) and shSIRT6+DCS (20 μM). Scale bar, 50 μm. (m, n) Quantification of total dendritic length and the branch number (m) and sholl analysis of dendritic morphology (n) of primary hippocampal neurons from the five groups (n = 20 neurons from 6-8 mice). (o) Representative single cell traces of intracellular spontaneous calcium activity of the five groups. (p) Calcium spike frequency and mean fluorescence intensity from the five groups (n = 6 fields from 3 independent experiments). (q, r) Representative WB images (q) and quantification (r) p-AKT/AKT, p-mTOR/mTOR and p-p70S6K1/p70S6K1 protein levels from the four groups. The data are shown as mean ± SEM. Two-tailed Student's t test in (a), (d); one-way ANOVA with Tukey's multiple comparisons test in (f), (i), (k), (m), (p), (r); two-

way ANOVA followed by Sidak's multiple comparisons test was used for multiple comparisons in (g), (n). *$p < 0.05$, **$p < 0.01$, ***$p < 0.001$, ns, not significant.

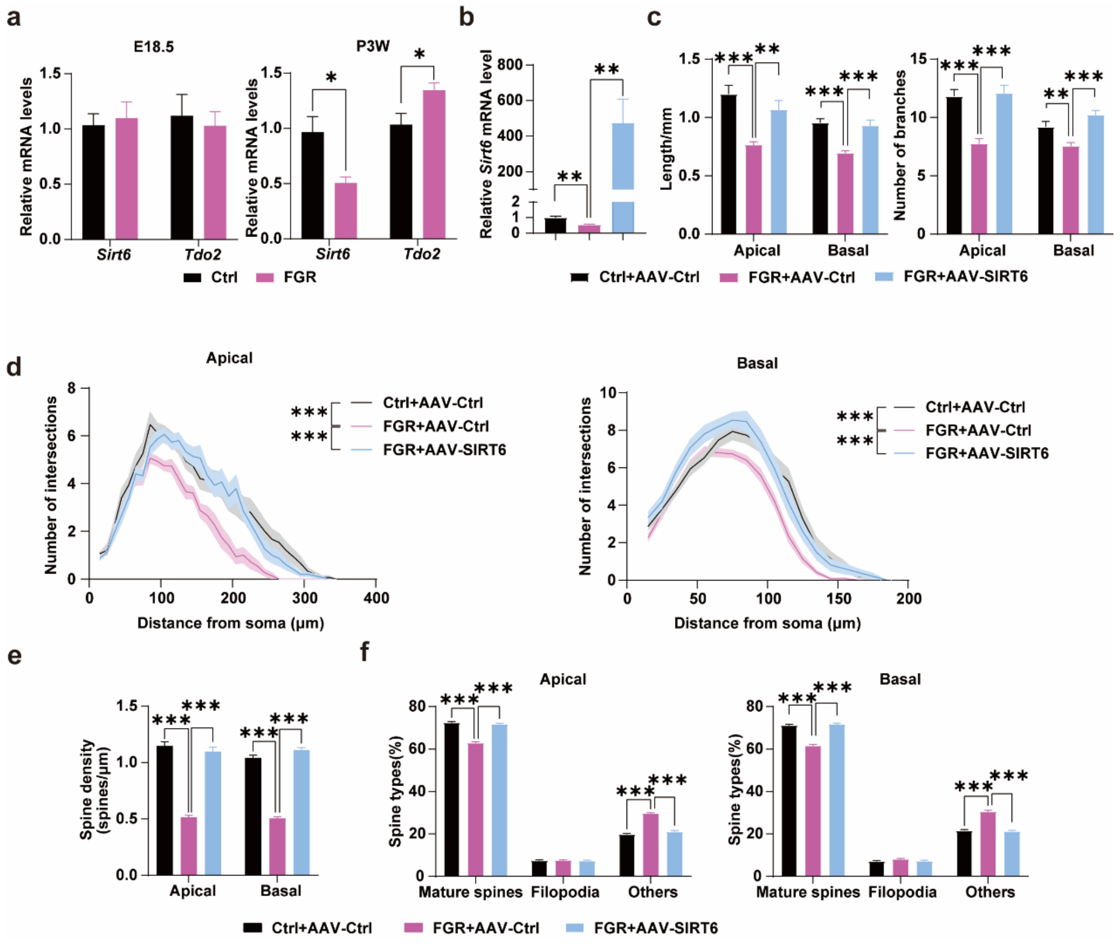

Supplementary Fig. 7 Restoration of SIRT6 expression rescues dendritic and synaptic deficits in FGR mice.

(a) qPCR analysis of *Sirt6* and *Tdo2* mRNA levels in the hippocampus of Ctrl and FGR mice in E18.5 (n = 5 mice per group) and P3W (n = 4 mice per group). (b) Quantitative PCR analysis of hippocampal *Sirt6* mRNA levels following AAV injection in Ctrl+AAV-Ctrl, FGR+AAV-Ctrl, and FGR+AAV-SIRT6 groups (n = 6 mice per group). (c) Quantification of the dendritic length and branch numbers in the apical and basal dendrites of hippocampal CA1 neurons from the three groups (n = 15 neurons from 3 mice per group). (d) Sholl analysis of dendritic complexity in in the apical and basal

dendrites of hippocampal CA1 neurons from three groups (n = 15 neurons from 3 mice per group). (e, f) Quantification of dendritic spine density (e) and distribution of spine types (f) in the apical and basal dendrites of CA1 pyramidal neurons from the three groups (n = 15 neurons from 3 mice per group). The data are shown as mean ± SEM. Two-tailed Student's t test in (a), (b); one-way ANOVA with Tukey's multiple comparisons test in (c), (e), (f); two-way ANOVA followed by Sidak's multiple comparisons test was used for multiple comparisons in (d). *$p < 0.05$, **$p < 0.01$, ***$p < 0.001$.

Supplementary Table 1. Primers list in this study.

Gene symbols	Primer sequences (5' to 3')	F/R
**Primer sets used in PCR**		
**Sirt6**	GCTAATGGGAACGAGACCAA	P1-F
	ACCCACCTCTCTCCCCTAAA	P2-R
	GCGTCCACTTCTCTTTCCTG	P3-R
**Cre**	CAGCATTGCTGTCACTTGGTC	F
	ATTTGCCTGCATTACCGGTCG	R
**Primer sets used in qRT-PCR**		
**Sirt1**	CTCTGAAAGTGAGACCAGTAGC	F
	TGTAGATGAGGCAAAGGTTCC	R
**Sirt2**	ACGATGGGCTGGATGAAAG	F
	TTTACCACGCTCTGACACTG	R
**Sirt3**	CGGCTCTATACACAGAACATCG	F
	CATCAGCCCATATGTCTTCCC	R
**Sirt4**	CTACAGGTCAGAAAAGGTGGG	F

	CGGACGAAATCAATGTGCTG	R
**Sirt5**	TCCCCACAAAGCAAGATCTG	F
	CGTTCGCAAAACACTTCCG	R
**Sirt6**	TTCAGCTAGAACGCATGGG	F
	TCTTACACTTGGGACATTCCTC	R
**Sirt7**	TGTGTTTAGGGTCCAGCTTG	F
	ACTTCCCATGGAGTTTCAGG	R
**Ido1**	CAATCAAAGCAATCCCCACTG	F
	AAAACGTGTCTGGGTCCAC	R
**Ido2**	CCTAAAGAGTTACCGTGAGCAG	F
	AACAAAAGGAATGGCAAGAGATC	R
**Tdo2**	CTCAAGGTGATAGCTCGGATG	F
	GAACTGTAGACTCTGGAAGCC	R
**Kat2**	ATGAATTACTCACGGTTCCTCAC	F
	AACATGCTCGGGTTTGGAGAT	R
**Kat4**	GGACCTCCAGATCCCATCCT	F
	GGTTTTCCGTTATCATCCCGGTA	R
**GR**	GGACCACCTCCCAAACTCTG	F
	GCTGTCCTTCCACTGCTCTT	R
**mTOR**	CACAAGGAGATCCGCATGGA	F
	GCGGATATCAGGGTCAGGAT	R
**p70S6K**	CACCTGTCAGCCCAGTCAAA	F
	CCGCTCACTGTCACATCCAT	R
**Raptor**	CTTCCAACAACACGAGGAGC	F
	TCTCCCGGTCAGTATCCCAG	R

**Actin**	CTGTATTCCCCTCCATCGTG	F
	GCCTCGTCACCCACATAG	R
**Primer sets used in vetor construction**		
**Gene symbols**	**Primer sequences (5' to 3')**	**F/R**
**shCtrl**	CCGGCCTAAGGTTAAGTCGCCCTCGCTCGAGCGAGGGCGACTTAACCTTAGGTTTTTG	F
	AATTCAAAAACGAGGGCGACTTAACCTTAGGCTCGAGCCTAAGGTTAAGTCGCCCTCG	R
**shSIRT6**	CCGGTGCGCATCCACGGCTACGTGGACTCGAGTCCACGTAGCCGTGGATGCGCATTTTTG	F
	AATTCAAAAATGCGCATCCACGGCTACGTGGACTCGAGTCCACGTAGCCGTGGATGCGCA	R
**shGR**	CCGGGGAACATGCTCAAGGTGATAGCTCGAGCTATCACCTTGAGCATGTTCCTTTTTG	F
	AATTCAAAAAGGAACATGCTCAAGGTGATAGCTCGAGCTATCACCTTGAGCATGTTCC	R
**Primer sets used in ChIP-qPCR**		
**Gene symbols**	**Primer sequences (5' to 3')**	**F/R**
**TDO2-1** (–1899 to –1782 bp)	AAATGTAATCTGACCCAGACCC	F
	CAATGGCCTGGTCGTTACTTA	R
**TDO2-2** (–1361 to –1221 bp)	TCTGAGAAACTTGAGGCTGAAG	F
	CTTTGTGTTCTGTTGAGCCAAG	R
**TDO2-3** (–827 to –628 bp)	CCACCCACTCCTGCATCAAT	F
	TCAACCAACCAGACCCCCTA	R
**TDO2-4** (–338 to –222 bp)	CCCTGCATTTCGTTTTGAGAG	F
	AGAACCTGTAGACCCCATATCC	R
**GR-1/ RNAPII-1**	ACTTTGTAGACCAGGCTGGC	F

(–1985 to –1891 bp)	TGAAAAACAAAAACAACAACAACAAAA	R
**GR-2/ RNAPII-2** (–1361 to –1221 bp)	TCTGAGAAACTTGAGGCTGAAG	F
	CTTTGTGTTCTGTTGAGCCAAG	R
**GR-3/ RNAPII-3** (–759 to –590 bp)	TGGGTGGCCCTATCCCTTAG	F
	GGGGAATTGGGAGTAGCCAC	R
**GR-4/ RNAPII-4** (–495 to –353 bp)	CCCCTCCTCTCTCCCTTCTAG	F
	GCAAAAGTTTAACTTCTCCCAGTAGT	R
**RPL23**	TGTGCGGTGGGTTATAGTTG	F
	TCTGCACAGTTGATCACAGC	R
**Negative control**	GCGGTCTCAAAAGCAAACAG	F
	GATCTACCTGTCTTTGCCTCC	R

Supplementary Table 2. Antibodies list in this study.

**Antibody**	**Catalognumber/manufacturer**	**Dilution**
**SIRT6**	ab62739, Abcam<br>ab191385, Abcam	1:1000 for WB<br>1:1000 for IF<br>4μg for ChIP
**GR**	ab2768, Abcam	4μg for ChIP
**RNAPII**	05-623, Millipore	4μg for ChIP
**KAT2**	A13090, ABclonal	1:1000 for WB<br>1:1000 for IF
**KAT4**	A6915, ABclonal	1:1000 for WB<br>1:1000 for IF
**ACTIN**	AC026, ABclonal	1:1000 for WB

**mCherry**	MB2013, Bioworld	1:1000 for IF
**CaMKIIα**	05-532, Sigma-Aldrich	1:500 for IF
**GFAP**	MAB360, Millipore	1:1000 for IF
**Ibal1**	011-27991, wako	1:500 for IF
**SOX2**	AF2018, R&D Systems	1:500 for IF
**GFP**	2955, Cell Signaling Technology	1:1000 for IF
**TDO2**	A13182, ABclonal	1:1000 for WB
**H3K9ac**	06-942, Millipore	1:1000 for WB<br>4μg for ChIP
**H3K56ac**	PA5-40101, Thermo Fisher	1:1000 for WB<br>5μg for ChIP
**H3**	ab1791, Abcam	1:1000 for WB
**p-AKT**	4060L, Cell Signaling Technology	1:1000 for WB
**AKT**	2920S, Cell Signaling Technology	1:1000 for WB
**p-mTOR**	AP0115, ABclonal	1:1000 for WB
**mTOR**	A2445, ABclonal	1:1000 for WB
**p-p70S6K1**	AP0564, ABclonal	1:1000 for WB
**p70S6K1**	A4898, ABclonal	1:1000 for WB
**MAP2**	ab32454, Abcam	1:5000 for IF
**GluA1**	A11643, ABclonal	1:1000 for WB
**PSD95**	36233S, Cell Signaling Technology<br>75-028, NeuroMab	1:1000 for WB<br>1:200 for IF
**Synapsin1**	574777, Millipore	1:1000 for IF
**Hoechst**	14533, Sigma-Aldrich	1:3000 for IF
**Mouse IgG HRP**	7076s, Cell Signaling Technology	1:2000 for WB

**Rabbit IgG HRP**	7074s, Cell Signaling Technology	1:2000 for WB
**Alexa Fluor 594 Donkey anti-Rabbit**	A21207, Invitrogen	1:1000 for IF
**Alexa Fluor 488 Donkey anti-Mouse**	A21202, Invitrogen	1:1000 for IF
**Alexa Fluor 488 Donkey anti-Goat**	A11055, Invitrogen	1:1000 for IF

Original Data file of Golgi staining

Figure 3a

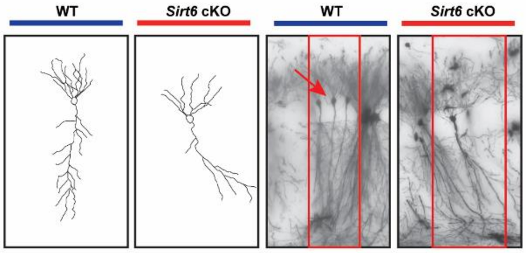

Figure 4d

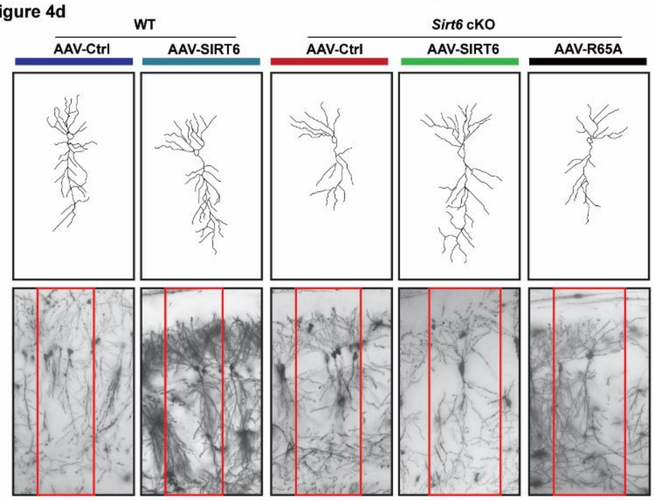

Figure 5i

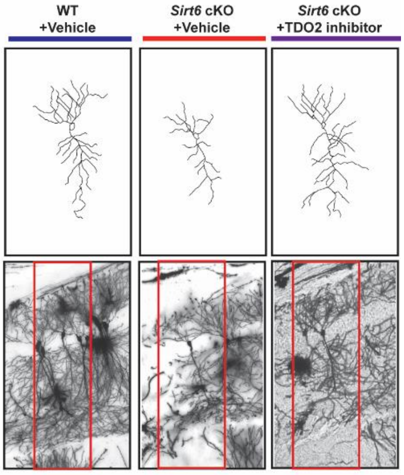

Figure 6j

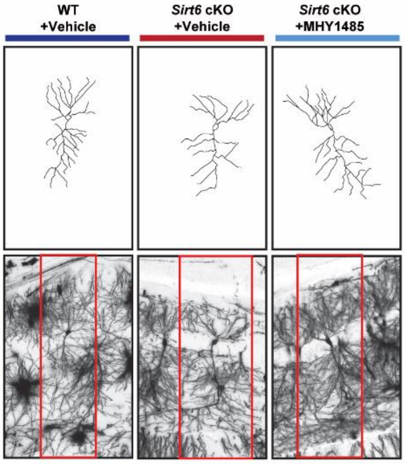

Figure 8i

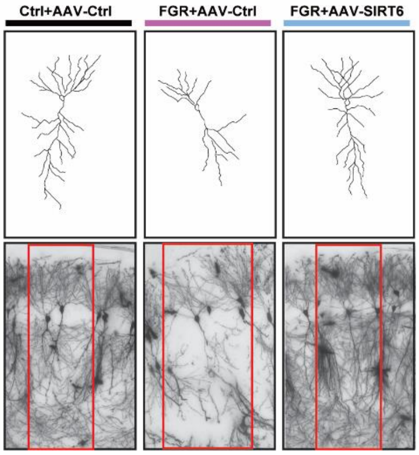

Supplement: Supplementary file 1 — Supplementary Information [file 12276_2026_1739_MOESM1_ESM.pdf]
